# SARS-CoV-2 spike-protein targeted serology test results and their association with subsequent COVID-19-related outcomes

**DOI:** 10.3389/fpubh.2023.1193246

**Published:** 2023-07-25

**Authors:** Harvey W. Kaufman, Stanley Letovsky, William A. Meyer, Laura Gillim, Magdalene M. Assimon, Carly A. Kabelac, John W. Kroner, Shannon L. Reynolds, Marcia Eisenberg

**Affiliations:** ^1^Quest Diagnostics^®^, Secaucus, NJ, United States; ^2^Labcorp^®^, Burlington, NC, United States; ^3^Aetion, Inc.^®^, New York, NY, United States

**Keywords:** SARS-CoV-2 spike antibody, SARS-CoV-2, COVID-19, immunocompromised conditions, immune protection

## Abstract

**Importance:**

In the absence of evidence of clinical utility, the United States' Centers for Disease Control and Prevention does not currently recommend the assessment of severe acute respiratory syndrome coronavirus-2 (SARS-CoV-2) spike-protein antibody levels. Clinicians and their patients, especially immunocompromised patients, may benefit from an adjunctive objective clinical laboratory measure of risk, using SARS-CoV-2 serology.

**Objective:**

The aim of this study is to estimate the association between SARS-CoV-2 spike-protein targeted antibody levels and clinically relevant outcomes overall and among clinically relevant subgroups, such as vaccine and immunocompetency statuses.

**Design:**

A retrospective cohort study was conducted using laboratory-based data containing SARS-CoV-2 antibody testing results, as well as medical and pharmacy claim data. SARS-CoV-2 testing was performed by two large United States-based reference clinical laboratories, Labcorp^®^ and Quest Diagnostics, and was linked to medical insurance claims, including vaccination receipt, through the HealthVerity Marketplace. Follow-up for outcomes began after each eligible individual's first SARS-CoV-2 semiquantitative spike-protein targeted antibody test, from 16 November 2020 to 30 December 2021.

**Exposures:**

Exposure is defined as having SARS-CoV-2 spike-protein targeted antibody testing.

**Main outcomes and measures:**

Study outcomes were SARS-CoV-2 infection and a serious composite outcome (hospitalization with an associated SARS-CoV-2 infection or all-cause death). Cox proportional hazards models were used to estimate hazard ratios (HRs) and 95% confidence intervals (CIs). Propensity score matching was used for confounding covariate control.

**Results:**

In total, 143,091 (73.2%) and 52,355 (26.8%) eligible individuals had detectable and non-detectable levels of SARS-CoV-2 spike-protein targeted antibodies, respectively. In the overall population, having detectable vs. non-detectable antibodies was associated with an estimated 44% relative reduction in SARS-CoV-2 subsequent infection risk (HR, 0.56; 95% CI 0.53–0.59) and an 80% relative reduction in the risk of serious composite outcomes (HR 0.20; 95% CI 0.15–0.26). Relative risk reductions were observed across subgroups, including among immunocompromised persons.

**Conclusion and relevance:**

Individuals with detectable SARS-CoV-2 spike-protein targeted antibody levels had fewer associated subsequent SARS-CoV-2 infections and serious adverse clinical outcomes. Policymakers and clinicians may find SARS-CoV-2 spike-protein targeted serology testing to be a useful adjunct in counseling patients with non-detectable antibody levels about adverse risks and reinforcing appropriate actions to mitigate such risks.

## Introduction

During the coronavirus disease-2019 (COVID-19) pandemic, severe acute respiratory syndrome coronavirus-2 (SARS-CoV-2) spike-protein targeted serology testing has played only a limited role in clinical decision-making, largely based on the advice from the United States Centers for Disease Control and Prevention (CDC) (1). CDC Guidance, updated on 16 December 2022, states, “Antibody testing is not currently recommended to assess for immunity to SARS-CoV-2 following COVID-19 vaccination or to assess the need for vaccination in an unvaccinated person” (2). Furthermore, the United States Food and Drug Administration (FDA) has not designated any Emergency Use Authorization (EUA) SARS-CoV-2 test for assessing individual immunity through antibody testing (The FDA reviews and responds to submissions from *in vitro* diagnostics manufacturers). Currently, the efficacy of detectable antibody levels against subsequent SARS-CoV-2 infection and adverse outcomes is incompletely understood. As a result, only a small number of studies have evaluated the risk of SARS-CoV-2 infection outcomes based on SARS-CoV-2 antibody testing, and none have evaluated this risk among subgroups of the population at the highest risk for severe adverse outcomes of SARS-CoV-2 infection, e.g., immunocompromised individuals (3–5).

The need for clinical guidelines for using SARS-CoV-2 serology testing at the individual level is most acute for immunocompromised persons and those with chronic medical conditions (6). These groups are at increased risk for serious adverse COVID-19-related outcomes, including hospitalization and death (7–10). Immunocompromised patients hospitalized with COVID-19 accounted for 12.2% of hospitalized SARS-CoV-2 infected patients but only 2.7% of the general population (11). Furthermore, immunocompromised persons are more likely to experience adverse COVID-19 outcomes, regardless of vaccination status (11). Having other chronic medical conditions and advanced age are associated with an increased vulnerability to adverse COVID-19 outcomes including age 65 years and older (6), diabetes, cardiovascular disease including hypertension, chronic kidney disease (12), and obesity (13, 14).

The objective of this study was to investigate if having detectable vs. non-detectable SARS-CoV-2 spike-protein targeted antibody levels was associated with a decreased risk of COVID-19-related adverse outcomes overall and among clinically relevant subgroups.

## Methods

### Data sources

Data were analyzed from HealthVerity-curated real-world sources in the United States, including de-identified closed medical and pharmaceutical claims, clinical laboratory data, COVID-19 vaccination records, and mortality records with services provided between 1 July 2020 and 28 February 2022.

### Study design and population

**Figure 1:**
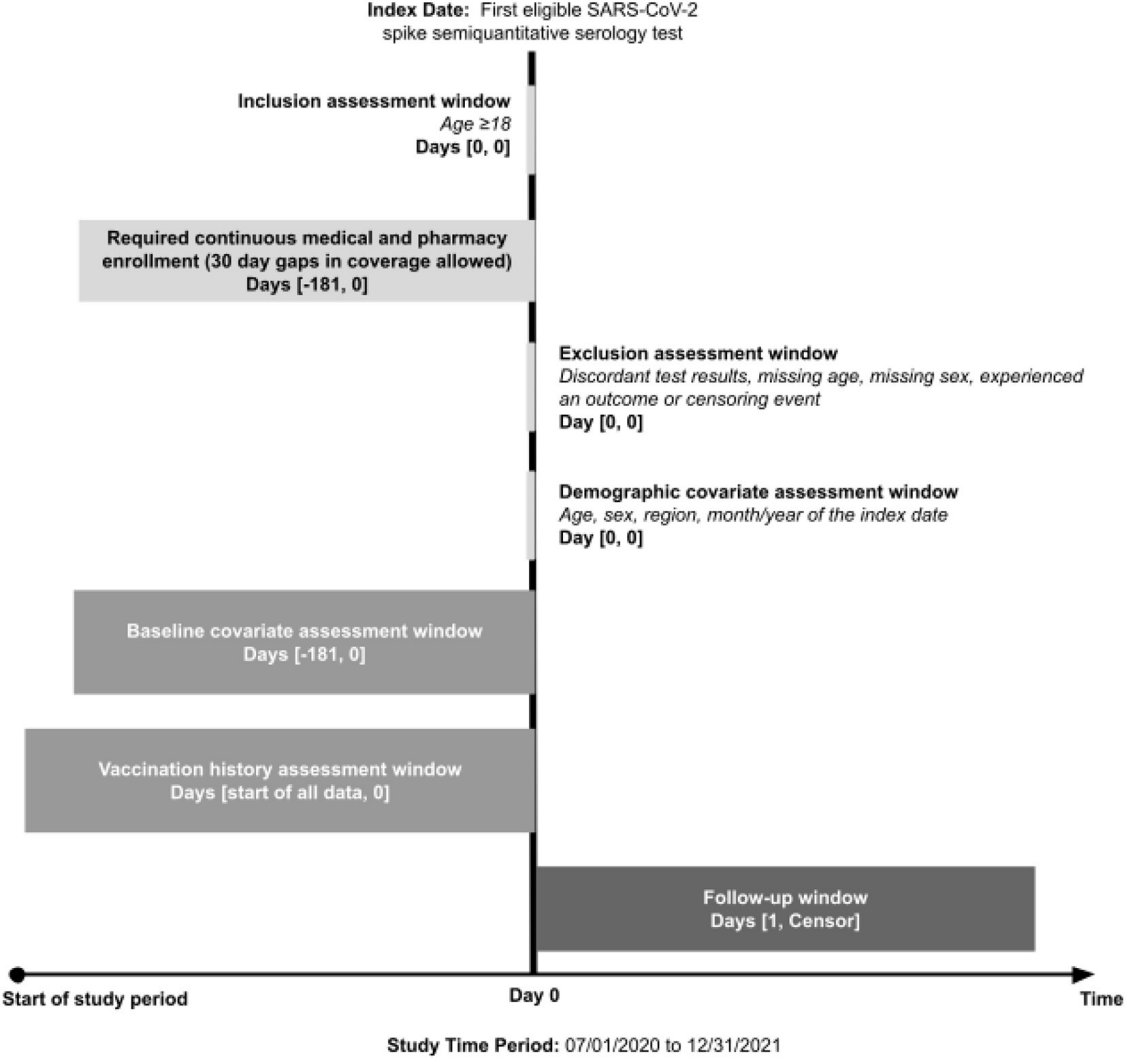
Study design. COVID-19, coronavirus disease 2019; SARS-CoV-2, severe acute respiratory syndrome coronavirus 2.

A retrospective cohort study was conducted to investigate the association between having detectable vs. non-detectable SARS-CoV-2 spike-protein targeted antibody levels and the occurrence of subsequent COVID-19-related outcomes. Figure 1 displays the flow diagram of the study cohort selection. Individuals entered the study cohort if their first eligible SARS-CoV-2 spike-protein targeted semiquantitative antibody test was performed between 16 November 2020 and 30 December 2021. Subjects were excluded if they met one or more of the following criteria: (1) lacked continuous insurance enrollment during 181 days prior to and including the antibody test date (allowing for 30-day gaps), (2) had discordant antibody test results, (3) were <18 years of age, (4) had missing age or sex information, or (5) experienced a study outcome or censoring event on the antibody test date.

### Exposure, outcomes, and covariates

The study individual's index date was defined as the date of the individual's first eligible SARS-CoV-2 spike-protein targeted semiquantitative antibody test. The exposure of interest was having detectable vs. non-detectable levels of SARS-CoV-2 spike protein antibody levels, as measured by commercially available semiquantitative assays. Four different FDA EUA assays, intended to identify individuals who may have developed an adaptive immune response to SARS-CoV-2, were in use by Labcorp and Quest Diagnostics during the study period: Siemens Healthcare Diagnostics, Inc ADVIA Centaur^®^ SARS-CoV-2 IgG (COV2G) and ADVIA Centaur/Atellica^®^ (sCOVG) assays, Roche Diagnostics Inc. Elecsys^®^ Anti-SARS-CoV-2 S, and DiaSorin Inc. LIAISON^®^ SARS-CoV-2 TrimericS IgG. The threshold for classifying people as having either detectable or non-detectable antibody levels differed across assays, and thus, test-specific levels were defined (Supplementary Table 1).

Outcomes of interest were as follows: (1) subsequent SARS-CoV-2 infection [i.e., positive polymerase chain reaction (PCR) or other nucleic acid amplification tests (NAATs)] and separately (2) a composite of serious events, hospitalization with an associated SARS-CoV-2 infection, or all-cause mortality (Supplementary Table 2). The follow-up period for outcome assessment started on the day after the index date, and outcomes were ascertained using medical claims, laboratory, and mortality data.

Baseline covariates included potential confounders and variables known to be strong risk factors for the study outcomes (15). Covariates of interest were identified prior to or on the index date and included demographic characteristics, skilled nursing facility or nursing home residence, the presence of an immunocompromising condition, and other clinical conditions associated with a heightened risk of severe COVID-19 (i.e., vulnerable medication conditions) and COVID-19 vaccination status (Supplementary Tables 3–4).

### Statistical analysis

All statistical analyses were performed using the Aetion Evidence Platform^®^ version 4.63 with R version 3.4.2. Baseline characteristics are described across individuals with detectable and non-detectable levels of antibodies against SARS-CoV-2 spike protein as count (%) for categorical variables and as mean ± standard deviation for continuous variables. Covariate distributions were compared between exposure groups using absolute standardized mean differences (ASDs). An ASD ≤0.10 indicates adequate covariate balance between groups (16).

An as-treated analytic approach was used to evaluate the association between having detectable vs. non-detectable levels of SARS-CoV-2 spike-protein targeted antibodies and the occurrence of COVID-19-related outcomes. Individuals were followed forward in historical time starting from the day after the index date until the occurrence of an outcome or censoring event. Censoring events included the following: (1) change in exposure status, (2) insurance disenrolment, and (3) study end (31 December 2021).

In primary analyses, 1:1 propensity score matching was used for confounding control. Nearest-neighbor matching without replacement was performed using a caliper of 1.0%. Cox proportional hazards models were used to estimate hazard ratios (HRs) and 95% confidence intervals (CIs) in both the unmatched and propensity score matched cohorts.

Given that associations may differ across clinically relevant subgroups, secondary subgroup analyses were conducted using analogous methods. Subgroups of interest included COVID-19 vaccination status subgroups (vaccinated and unvaccinated individuals) and health status subgroups (immunocompromised, vulnerable, and other healthy individuals) (Supplementary Table 5).

Finally, to understand whether higher SARS-CoV-2 spike-protein antibody levels may be associated with subsequent protection against SARS-CoV-2 infection, additional analyses were conducted among individuals with detectable levels of SARS-CoV-2 antibodies. In these analyses, all semiquantitative antibody test result values were converted to a common scale, WHO binding antibody units (BAUs; Supplementary Table 6) (16–18). Higher antibody levels were defined as having test results of ≥250 BAU/ml and lower antibody levels as having detectable test results of <250 BAU/ml, based on studies supporting this general threshold (19–21). EQUATOR Reporting Guidelines were followed.

## Results

A total of 1,798,606 people had a SARS-CoV-2 spike-protein targeted semiquantitative serology test from 16 November 2020 to 30 December 2021 (Supplementary Figure 1). Lack of baseline insurance enrollment excluded 1,580,452 individuals, and an additional 22,708 persons were excluded for other reasons. Therefore, the study cohort included a total of 195,446 individuals: 143,091 (73.2%) individuals with detectable levels and 52,355 (26.8%) individuals with non-detectable levels of semiquantitative SARS-CoV-2 spike-protein targeted antibodies. Overall, the study cohort had a mean age of 50.6 years, was 60.2% female, and was most commonly from the Northeastern (39.0%) and Southern (36.4%) regions of the United States. Only 24.9% of individuals had a record of receiving at least one dose of a COVID-19 vaccine. Of study individuals with any documented vaccination prior to exposure (SARS-CoV-2 spike-protein antibody testing), 95.5% (46,373 of 48,711) had a detectable antibody. In contrast, only 65.9% (96,718 of 146,735) of study individuals without documented vaccination prior to exposure had detectable antibody levels. Having an immunocompromising medical condition or another vulnerable medical condition associated with a heightened risk of severe COVID-19 was present in 7.0 and 45.9% of the cohort, respectively.

Table 1 shows the baseline characteristics of the study cohort stratified by the exposure groups. Before propensity score matching, baseline covariates were generally well-balanced between exposure groups (ASD ≤0.10), with some exceptions (e.g., age, region of the United States, year/season of cohort entry, and COVID-19 vaccination status). After propensity score matching, all baseline covariates were well-balanced between exposure groups, indicating adequate control of measured confounders.

**Table 1:** Characteristics of individuals with detectable and non-detectable SARS-CoV-2 spike-protein targeted antibody levels.

**Characteristic**	**Unmatched** ^**a**^			**Matched** ^**a**^		
	**Detectable antibody level** ***n** =* **143,091**	**Non-detectable antibody level** ***n** =* **52,355**	**Std diff** ^b^	**Detectable antibody level** ***n** =* **51,807**	**Non-detectable antibody level** ***n** =* **51,807**	**Std diff** ^b^
**Year/season of index** ^**c**^			0.30			0.02
Winter 2020–2021	1,224 (0.9%)	577 (1.1%)		511 (1.0%)	572 (1.1%)	
Spring 2021	22,683 (15.9%)	12,828 (24.5%)		12,323 (23.8%)	12,437 (24.0%)	
Summer 2021	42,750 (29.9%)	17,433 (33.3%)		17,215 (33.2%)	17,285 (33.4%)	
Fall 2021	57,416 (40.1%)	17,600 (33.6%)		17,842 (34.4%)	17,596 (34.0%)	
Winter 2021	19,018 (13.3%)	3,917 (7.5%)		3,916 (7.6%)	3,917 (7.6%)	
**Age (years)**	51.6 ± 15.6	48.0 ± 14.5	0.24	47.9 ± 14.7	48.1 ± 14.5	0.01
**Female**	86,764 (60.6%)	30,983 (59.2%)	0.03	30,962 (59.8%)	30,773 (59.4%)	0.01
**Region**			0.28			0.01
Midwest	11,755 (8.2%)	6,717 (12.8%)		6,371 (12.3%)	6,459 (12.5%)	
South	51,701 (36.1%)	19,411 (37.1%)		19,483 (37.6%)	19,374 (37.4%)	
West	19,448 (13.6%)	10,214 (19.5%)		9,948 (19.2%)	9,961 (19.2%)	
Northeast	60,166 (42.0%)	16,007 (30.6%)		16,004 (30.9%)	16,007 (30.9%)	
Other or unknown	21 (0.0%)	6 (0.0%)		1 (0.0%)	6 (0.0%)	
**SNF or nursing home utilization**	1,117 (0.8%)	303 (0.6%)	0.03	196 (0.4%)	299 (0.6%)	0.03
**Had** **≥1 immunocompromising condition**	10,467 (7.3%)	3,263 (6.2%)	0.04	3,179 (6.1%)	3,192 (6.2%)	0.00
**Had** **≥1 vulnerable condition**	69,032 (48.2%)	20,671 (39.5%)	0.18	20,524 (39.6%)	20,572 (39.7%)	0.00
**COVID-19 vaccination status**			0.77			0.00
Fully vaccinated plus a booster	1,067 (0.7%)	30 (0.1%)		33 (0.1%)	30 (0.1%)	
Fully vaccinated	35,415 (24.7%)	1,726 (3.3%)		1,709 (3.3%)	1,726 (3.3%)	
Partially vaccinated	9,891 (6.9%)	582 (1.1%)		590 (1.1%)	582 (1.1%)	
Unvaccinated	96,718 (67.6%)	50,017 (95.5%)		49,475 (95.5%)	49,469 (95.5%)	

a Values presented as number (%) for categorical variables and as mean ± standard deviation for continuous variables.

b A standardized difference >0.10 represents a meaningful imbalance between exposure groups.

c Winter 2020–2021 was defined as 1 December 2022 to 28 February 2021. Spring 2021 was defined as 1 March 2021 to 31 May 2021. Summer 2021 was defined as 1 June 2021 to 31 August 2021. Fall 2021 was defined as 1 September 2021 to 30 November 2021. Winter 2021 was defined as 1 December 2021 to 31 December 2021.

COVID-19, coronavirus disease 2019; SNF, SARS-CoV-2, severe acute respiratory syndrome coronavirus 2; skilled nursing facility; std diff, standardized different.

### Primary analyses

**Figure 2:**
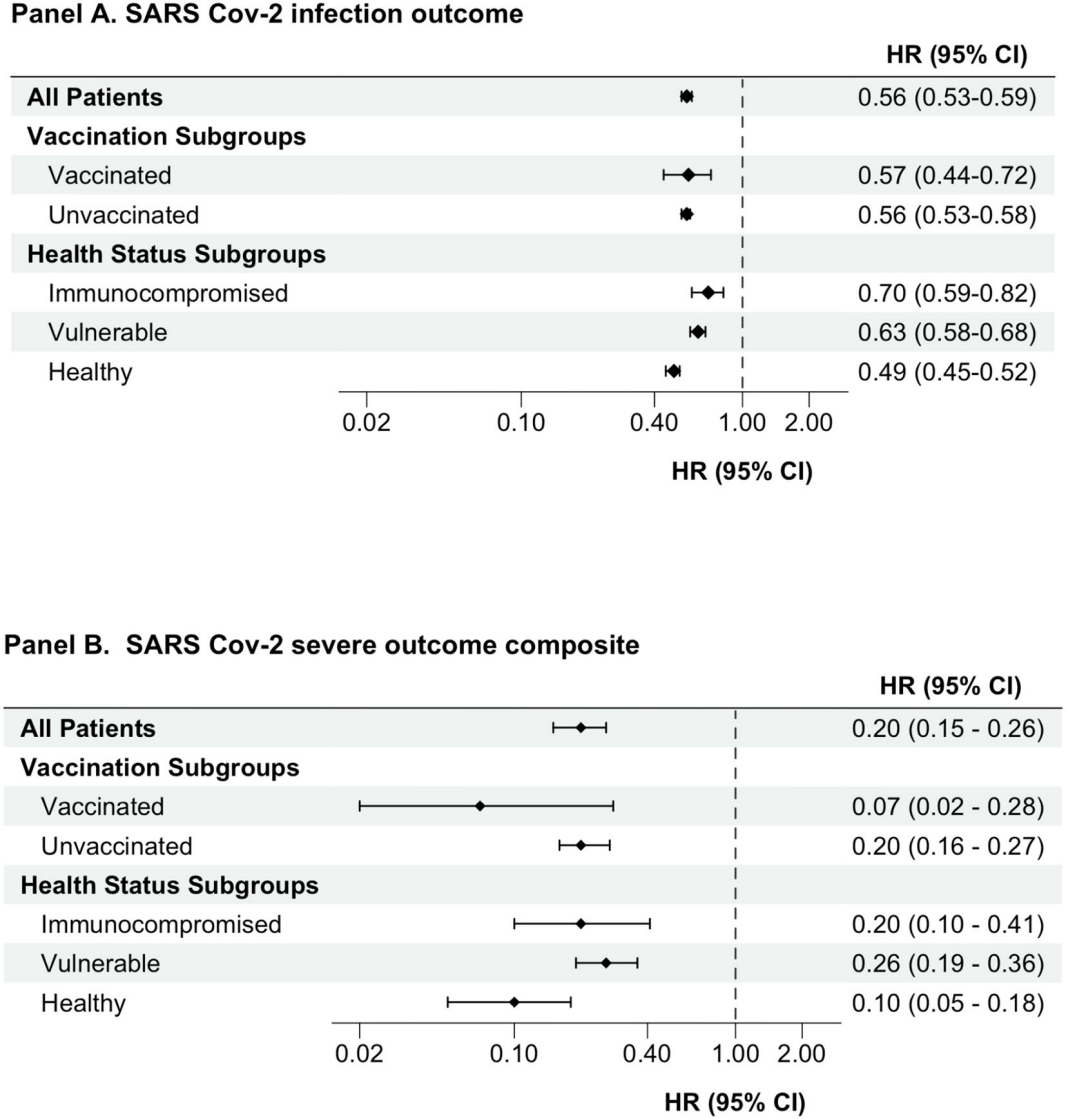
Association between having detectable versus non-detectable SARS-CoV-2 spike-protein targeted antibody levels and outcomes. An as-treated analytic approach was used for all analyses. Cox proportional hazards models were used to estimate hazard ratios (CI) comparing individuals with detectable versus non-detectable levels of semi quantitative antibodies against SARS-CoV-2 spike protein. HRs presented is for propensity score-matched cohort. CI, confidence interval; HR, hazard ratio; SARS-CoV-2, severe acute respiratory syndrome coronavirus 2.

During follow-up, a total of 10,735 subsequent SARS-CoV-2 infections occurred in the unmatched cohort: 6,392 events at an incidence rate of 141.1 events per 1,000 person-years in the detectable antibody group compared to 4,343 events at an incidence rate of 229.6 per 1,000 person-years in the non-detectable antibody group. Additionally, 575 serious outcomes (hospitalization with COVID-19 or all-cause mortality) occurred during follow-up: 221 events at an incidence rate of 4.8 per 1,000 person-years in the detectable antibody group compared to 354 events at an incidence rate of 18.1 per 1,000 person-years in the non-detectable antibody group. After propensity score matching, having detectable vs. non-detectable levels of SARS-CoV-2 spike-protein targeted antibodies was associated with a lower risk of subsequent SARS-CoV-2 infection (HR, 0.56; 95% CI 0.53–0.59) and the serious composite outcomes of hospitalization with COVID-19 or all-cause mortality (HR 0.20; 95% CI 0.15–0.26) (Figure 2).

### Subgroup analyses

Analyses of clinically relevant subgroups produced results analogous to our primary study findings. In both the unvaccinated and vaccinated subgroups, people with detectable SARS-CoV-2 spike-protein targeted antibody levels had a lower risk of subsequent SARS-CoV-2 infection and the serious outcomes composites compared to people with non-detectable antibody levels (Table 2 and Figure 2). Similarly, in the subgroups of immunocompromised, vulnerable, and otherwise healthy persons, having detectable antibody levels was associated with a lower risk of subsequent SARS-CoV-2 infection and experiencing serious composite outcomes of hospitalization with COVID-19 or all-cause mortality (Table 2 and Figure 2). The HR (95% CI) for subsequent SARS-CoV-2 infection in the groups with detectable SARS-CoV-2 spike-protein antibody levels (referent groups had non-detectable antibody levels) was 0.56 (0.53–0.59), 0.70 (0.59–0.82), 0.63 (0.58–0.68), and 0.49 (0.45–0.52) for the overall study cohort, immunocompromised, vulnerable, and other health groups, respectively. The HR (95% CI) for the serious composite outcomes in the groups with detectable SARS-CoV-2 spike-protein antibody levels (referent groups had non-detectable antibody levels) was 0.20 (0.15–0.26), 0.20 (0.10–0.41), 0.26 (0.19–0.36), and 0.10 (0.05–0.18) for the overall study cohort, immunocompromised, vulnerable, and other health groups, respectively.

**Table 2:** Association between having detectable vs. non-detectable SARS-CoV-2 spike-protein targeted antibody levels and outcomes, overall and in COVID-19 vaccination subgroups and in health status subgroups.

**SARS-CoV-2 infection, in COVID-19 vaccination subgroups**				
**Exposure**	**n**	**No. events (rate per 1,000 person years)**	**Unmatched cohort HR (95% CI)**	**Matched cohort HR (95% CI)**
**Overall**				
Non-detectable antibody levels	52,355	4,343 (229.6)	1.00 (ref.)	1.00 (ref.)
Detectable antibody levels	143,091	6,392 (141.1)	0.61 (0.59–0.64)	0.56 (0.53–0.59)
**Vaccinated**				
Non-detectable antibody levels	2,338	173 (236.7)	1.00 (ref.)	1.00 (ref.)
Detectable antibody levels	46,373	2,109 (159.5)	0.67 (0.57–0.78)	0.57 (0.44–0.72)
**Unvaccinated**				
Non-detectable antibody levels	50,017	4,170 (229.3)	1.00 (ref.)	1.00 (ref.)
Detectable antibody levels	96,718	4,283 (133.6)	0.58 (0.55–0.60)	0.56 (0.53–0.58)
**Hospitalization with COVID-19 or all-cause mortality, in COVID-19 vaccination subgroups**				
**Exposure**	**n**	**No. events (rate per 1,000 person years)**	**Unmatched cohort HR (95% CI)**	**Matched cohort HR (95% CI)**
**Overall**				
Non-detectable antibody levels	52,355	354 (18.1)	1.00 (ref.)	1.00 (ref.)
Detectable antibody levels	143,091	221 (4.8)	0.26 (0.22–0.31)	0.20 (0.15–0.26)
**Vaccinated**				
Non-detectable antibody levels	2,338	28 (37.2)	1.00 (ref.)	1.00 (ref.)
Detectable antibody levels	46,373	79 (5.9)	0.16 (0.10–0.24)	0.07 (0.02–0.28)
**Unvaccinated**				
Non-detectable antibody levels	50,017	326 (17.3)	1.00 (ref.)	1.00 (ref.)
Detectable antibody levels	96,718	142 (4.3)	0.25 (0.20–0.30)	0.20 (0.16–0.27)
**SARS-CoV-2 Infection, in health status subgroups**				
**Exposure**	**n**	**No. events (rate per 1,000 person years)**	**Unmatched cohort HR (95% CI)**	**Matched cohort HR (95% CI)**
**Overall**				
Non-detectable antibody levels	52,355	4,343 (229.6)	1.00 (ref.)	1.00 (ref.)
Detectable antibody levels	143,091	6,392 (141.1)	0.61 (0.59–0.64)	0.56 (0.53–0.59)
**Immunocompromised**				
Non-detectable antibody levels	3,263	323 (289.2)	1.00 (ref.)	1.00 (ref.)
Detectable antibody levels	10,467	642 (193.2)	0.67 (0.59–0.77)	0.70 (0.59–0.82)
**Vulnerable**				
Non-detectable antibody levels	18,575	1,651 (252.7)	1.00 (ref.)	1.00 (ref.)
Detectable antibody levels	62,139	3,030 (154.3)	0.61 (0.57–0.65)	0.63 (0.58–0.68)
**Other healthy**				
Non-detectable antibody levels	30,517	2,369 (210.3)	1.00 (ref.)	1.00 (ref.)
Detectable antibody levels	70,485	2,720 (121.8)	0.58 (0.55–0.61)	0.49 (0.45–0.52)
**Hospitalization with COVID-19 or all-cause mortality, in health status subgroups**				
**Exposure**	**n**	**No. events (rate per 1,000 person years)**	**Unmatched cohort HR (95% CI)**	**Matched cohort HR (95% CI)**
**Overall**				
Non-detectable antibody levels	52,355	354 (18.1)	1.00 (ref.)	1.00 (ref.)
Detectable antibody levels	143,091	221 (4.8)	0.26 (0.22–0.31)	0.20 (0.15–0.26)
**Immunocompromised**				
Non-detectable antibody levels	3,263	42 (35.9)	1.00 (ref.)	1.00 (ref.)
Detectable antibody levels	10,467	39 (11.4)	0.31 (0.20–0.49)	0.20 (0.10–0.41)
**Vulnerable**				
Non-detectable antibody levels	18,575	194 (28.6)	1.00 (ref.)	1.00 (ref.)
Detectable antibody levels	62,139	154 (7.6)	0.26 (0.21–0.33)	0.26 (0.19–0.36)
**Other healthy**				
Non-detectable antibody levels	30,517	118 (10.1)	1.00 (ref.)	1.00 (ref.)
Detectable antibody levels	70,485	28 (1.2)	0.12 (0.08–0.18)	0.10 (0.05–0.18)

An as-treated analytic approach was used for all analyses. Cox proportional hazards models were used to estimate hazard ratios (HR) [95% confidence intervals (CI)].

CI, confidence interval; COVID-19, coronavirus disease 2019; HR, hazard ratio; ref., referent; SARS-CoV-2, severe acute respiratory syndrome coronavirus 2.

### Additional analyses

**Figure 3:**
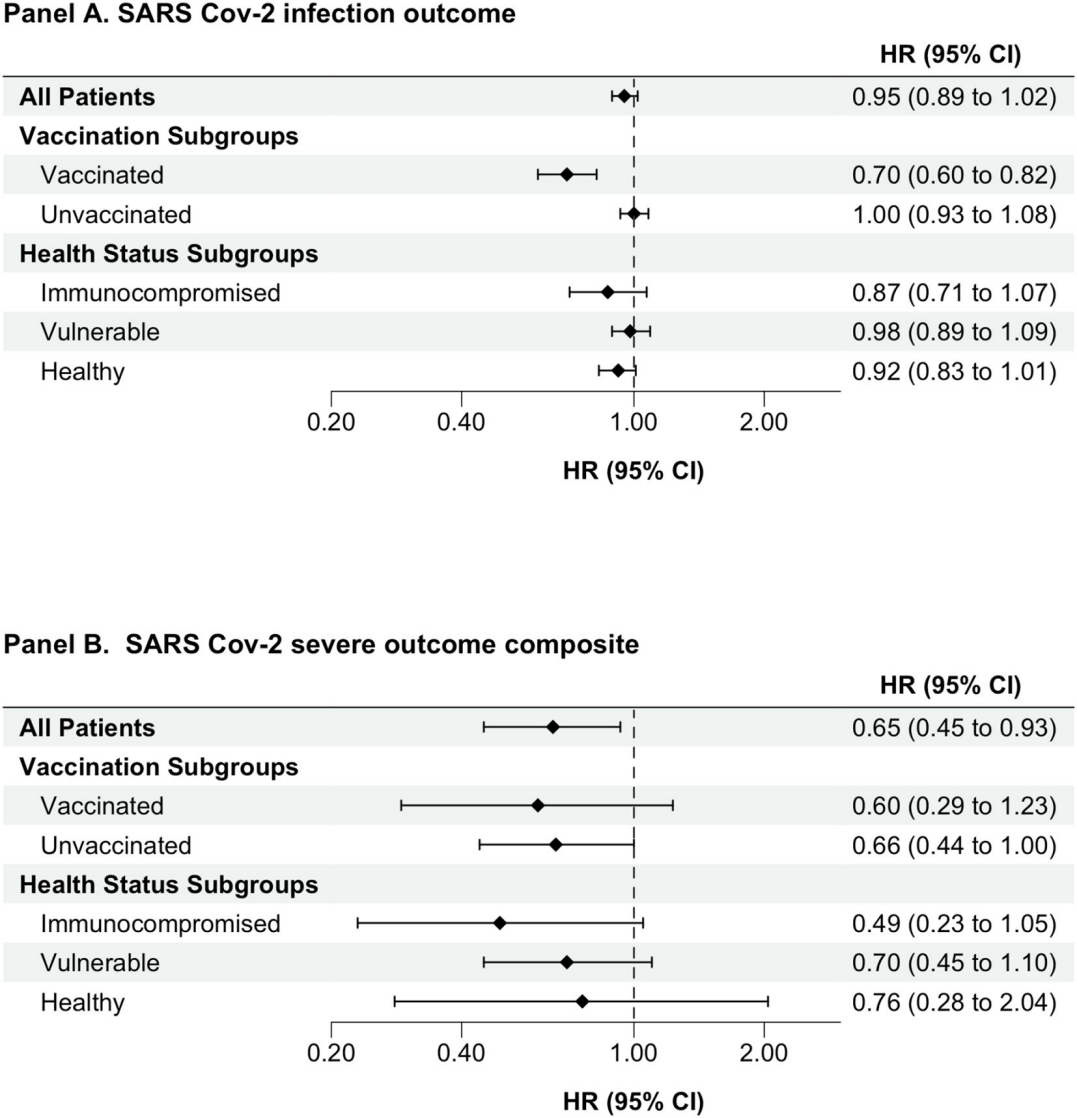
Association between having higher (≥250 BAU/mL) versus lower (< 250 BAU/mL) SARS-CoV-2 spike-protein antibody level comparison. An as-treated analytic approach was used for all analyses. Cox proportional hazards models were used to estimate hazard ratios (CI) comparing individuals with detectable versus non-detectable levels of semi quantitative antibodies against SARS-CoV-2 spike protein. HRs presented is for propensity score-matched cohort. BAU/ml, binding antibody units per milliliter; CI, confidence interval; HR, hazard ratio; SARS-CoV-2, severe acute respiratory syndrome coronavirus-2.

Among the group of individuals who had detectable levels of SARS-CoV-2 spike-protein targeted antibodies (Supplementary Figure 2 and Supplementary Table 5), those with higher (>250 BAU/ml) vs. lower (< 250 BAU/ml) antibody levels had a lower risk of serious outcomes (after propensity score matching HR, 0.65, 95% CI 0.45–0.93). Similar associational trends were seen within subgroups, but HR estimates were imprecise (Supplementary Table 7 and Figure 3).

## Discussion

Healthcare providers seek guidance on how to evaluate an individual's SARS-CoV-2 risk, especially for those with high-risk conditions, i.e., immunocompromised and vulnerable patients. Kaufman et al. have postulated that SARS-CoV-2 spike-protein targeted serology test results may be clinically useful, notably among these high-risk individuals concerning the subsequent risk of adverse outcomes (22). This position was based on a literature review that revealed (1) individuals at increased risk for severe outcomes following SARS-CoV-2 infection were less likely to develop a robust antibody response following infection and vaccination (23) and (2) studies showing that people with non-detectable or low levels of SARS-CoV-2 spike-protein targeted antibodies were more likely to have subsequent adverse events, i.e., hospitalization with COVID-19 and death, than those with higher levels (8, 24). Furthermore, SARS-CoV-2 spike-targeted antibody titer levels correlate with protection against subsequent SARS-CoV-2 infection or reinfection (25). This study confirms the two prior key findings by demonstrating that people with detectable SARS-CoV-2 spike-targeted antibody levels, as well as those with higher vs. lower detectable antibody levels, have a lower risk of COVID-19 serious outcomes in overall and adds novel findings among subgroups of patients at increased risk of SARS-CoV-2 infection (7–10).

These observations are consistent with other studies that have demonstrated similar associations between specific medical conditions and SARS-CoV-2 spike-protein targeted serology results and between serology results and subsequent outcomes (3–5). A recent study of cancer vs. non-cancer (control) patients in the United Kingdom showed that detectable levels of SARS-CoV-2 spike-protein targeted antibodies were associated with protection against subsequent SARS-CoV-2 infection and serious outcomes (26). Among patients with cancer, a non-detectable vaccine antibody response was associated with more than three times the risk of subsequent SARS-CoV-2 infection and more than six times the risk of a COVID-19-associated hospitalization. The authors suggest that patients with non-detectable antibody levels may benefit from additional vaccine doses, prophylactics, and early treatment.

In the United States, the three COVID-19 vaccines available during the study period were manufactured by BioNtech/Pfizer, Janssen/Johnson & Johnson, and Moderna. SARS-CoV-2 vaccination effectively reduced serious COVID-19 outcome events even though the benefit of vaccination is generally less effective among immunocompromised persons as compared to immunocompetent individuals (27). In this study, 24.9% (48,711 of 195,446) of individuals received at least one vaccine dose. In the matched cohort, the HR for subsequent infection among those with detectable vs. non-detectable SARS-CoV-2 spike-protein targeted antibody levels was 0.56 (95% CI 0.53–0.58) and 0.57 (95% CI 0.44–0.72) for the vaccinated and unvaccinated groups, respectively. In the matched cohort of this study, the HR for serious composite outcomes of hospitalization with COVID-19 or all-cause mortality among those with detectable vs. non-detectable SARS-CoV-2 spike-protein targeted antibody levels was 0.07 (95% CI 0.02–0.28) and 0.20 (95% CI 0.16–0.27) for the vaccinated and unvaccinated groups, respectively. Although the CI are overlapping, this observation supports the clinical value of having detectable antibodies while still recognizing that other immune mechanisms of protection are likely involved in the protection against SARS-CoV-2 infection and COVID-19 outcomes. Furthermore, the direction of the HR supports the concept that vaccination was likely useful in reducing serious outcomes.

Future studies may be useful in delineating the interplay of humoral and cellular immune system components in protection against SARS-CoV-2 infection and its consequences. Tixagevimab/cilgavimab was the only combination therapeutic authorized to date by both the FDA [effective 8 December 2021, revoked 26 January 2023 (28)] and the European Medicine Agency (EMA) (effective 25 March 2022) for pre-exposure prophylaxis of COVID-19. This was especially relevant in the population with immunocompromising conditions who fail to mount a detectable antibody response after multiple vaccine doses. The authors of the primary tixagevimab/cilgavimab study note, “The limitations of our trial include the low number of events in smaller but important subgroups, including immunocompromised persons, so that efficacy in these groups could not be estimated” (29). The FDA subsequently recommended high dosing of tixagevimab/cilgavimab after a significant number of patients in the immunosuppressed group were found to have breakthrough infections (30). Young-Xu et al. at Veteran Affairs Healthcare Systems, found that compared to 251,756 propensity-matched immunocompromised or at-risk historical controls, 1,848 tixagevimab/cilgavimab-treated patients had a lower incidence of SARS-CoV-2 infection, COVID-19 hospitalization, and all-cause mortality (31). Until newer effective prophylactic drugs are approved, respiratory tract masking may be especially valuable within the high-risk population, e.g., with immunocompromising conditions, when visiting healthcare facilities where there are potentially SARS-CoV-2-infected patients (32). The CDC suggests that masks can provide an extra level of protection against SARS-CoV-2 infection and its resulting severe events (6). Early antiviral treatments may be beneficial as well, especially in the immunocompromised population (33). Again, the effectiveness of these multiple infection mitigation measures with current and future SARS-CoV-2 variants is worthy of investigation. Given our findings among immunocompromised persons, the study's findings support the application of these suggested COVID-19 mitigation measures in high-risk populations, particularly those who are SARS-CoV-2 seronegative. Additional studies may indicate the specific potential benefit of additional vaccine dosing and other mitigation efforts to reduce the risk of adverse outcomes in individuals with non-detectable SARS-CoV-2 antibody levels.

The strengths of this study include the use of a large-scale real-world database with information aggregated from diverse sources, inclusive of multiple laboratory antibody test methods, analysis of subsequent SARS-CoV-2 infections, COVID-19 hospitalizations and all-cause mortality, and multivariate modeling with confounding control. Tracking changes in semiquantitative SARS-CoV-2 spike-protein targeted antibody levels over time within an at-risk individual may provide insights into the durability of the antibody response and assist in determining the subsequent risk of infection (5). Alternative assays that measure antibody neutralization of novel spike protein(s) or cellular-based adaptive immunity assays are being studied for their associated clinical utility but they are not yet widely commercially available (34). Of note, this study included SARS-CoV-2 spike-protein targeted antibody tests and did not include rapid antigen tests or nucleocapsid antibody tests. Differences in antibody generation post-infection have been observed with SARS-CoV-2 nucleocapsid antibody tests. SARS-CoV-2 Omicron variants impacted the performance of some NAAT assay methods. The FDA updates the few assay methods that are adversely affected by viral mutations (35). None of those SARS-CoV-2 NAAT assay methods were used in this study. Therefore, SARS-CoV-2 variants had no reported impact on the performance of the SARS-CoV-2 spike protein targeted antibody or NAAT testing in this study.

The study had some limitations. First, the evaluation time period was early in the pandemic (November 2020 to December 2021) and included a portion of the time when COVID-19 vaccines were not yet widely adopted. Similarly, home laboratory testing that identifies SARS-CoV-2 infections was not captured though also not yet common during the study period. Second, information on the medical reason for the requested SARS-CoV-2 serology testing was unavailable. Third, the COVID-19 infection outcome was largely driven by infections identified in the health insurance claims data. SARS-CoV-2 PCR/NAAT tests performed by LabCorp and Quest Diagnostics, although substantial in aggregate number, represent less than 20% of all total SARS-CoV-2 PCR/NAAT conducted in the United States during the study period (36–38). Finally, deaths were infrequent, precluding studying associations between SARS-CoV-2 antibody levels and mortality alone.

In summary, this large United States-based real-world evidence-based study utilized linked medical claims and clinical laboratory data to examine associations between SARS-CoV-2 spike-protein antibody levels and clinical outcomes. The study demonstrated that people with detectable levels of SARS-CoV-2 spike-protein targeted antibodies had a lower risk of subsequent SARS-CoV-2 infections and serious composite outcomes (hospitalization with an associated SARS-CoV-2 infection or all-cause mortality). This observed effect was seen in the overall population and also within clinically relevant subgroups, including the immunocompromised population. Analyses of individuals with detectable antibodies >250 vs. < 250 BAU/ml generated directionally consistent results, albeit with a less potent magnitude of effect. Thus, federal policymakers and clinicians may find SARS-CoV-2 spike-protein targeted serology testing to be a useful adjunct in counseling immunocompromised persons and other higher at-risk individuals about adverse outcomes and apply appropriate actions to mitigate such risks.

## Data availability statement

The raw data supporting the conclusions of this article will be made available by the authors, without undue reservation.

## Author contributions

All authors were involved in study design, analysis, interpretation of data, writing of the manuscript, and the decision to submit the manuscript for publication. All authors contributed to the article and approved the submitted version.
