# Supplementary material for: SARS-CoV-2 spike-protein targeted serology test results and their association with subsequent COVID-19-related outcomes
Source: Front Public Health. 2023 Jul 25;11:1193246. doi: 10.3389/fpubh.2023.1193246 (PMC10407563; doi:10.3389/fpubh.2023.1193246)
Supplement: Supplementary file 1 [file Data_Sheet_1.pdf]

Supplemental Tables and Figures:

Supplemental Table 1

Supplemental Table 2

Supplemental Table 3

Supplemental Table 4

Supplemental Table 5

Supplemental Table 6

Supplemental Figure 1

Supplemental Figure 2

Supplemental Table 7

----

eFigure 1. Flow diagram depicting study cohort assembly for the detectable versus non-detectable SARS-CoV-2 spike-protein antibody comparison

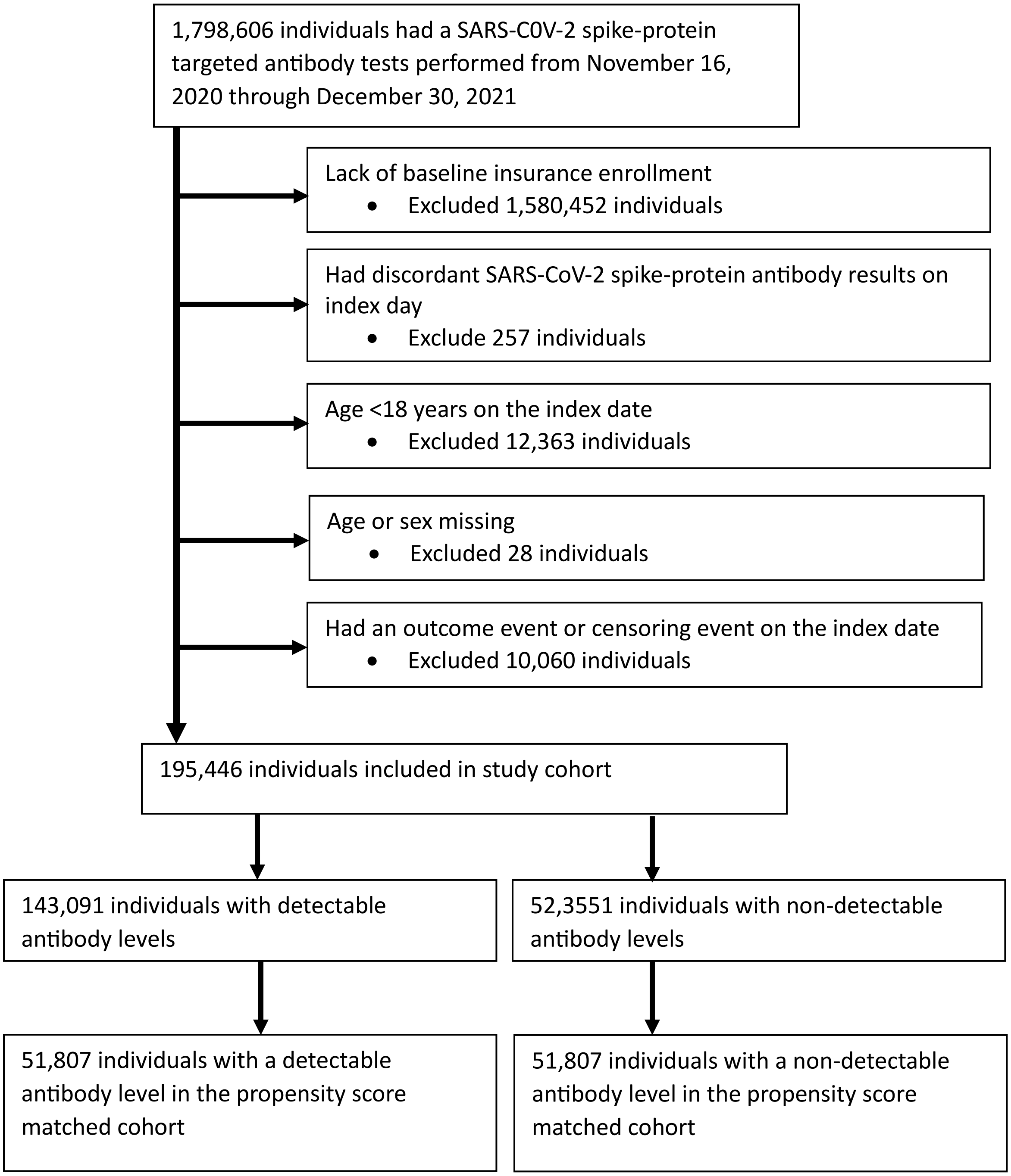

Abbreviation: SARS-CoV-2, severe acute respiratory syndrome coronavirus 2.

eFigure 2. Flow diagram depicting study cohort assembly for the higher (≥250 BAU/mL) versus lower (<250 BAU/mL) SARS-CoV-2 spike-protein antibody level comparison

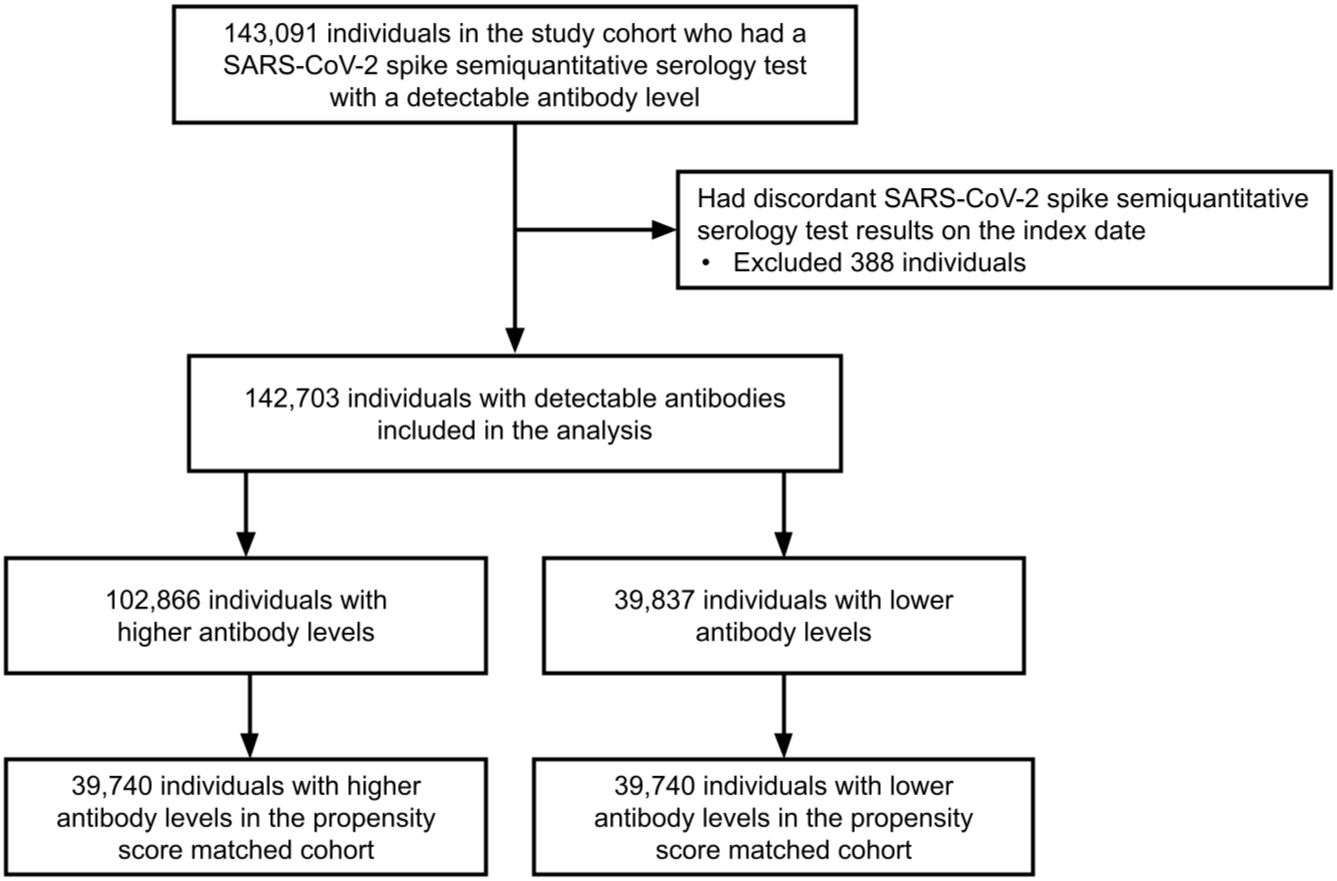

Abbreviations: BAU/ml, binding antibody units per milliliter; SARS-CoV-2, severe acute respiratory syndrome coronavirus 2.

eTable 1. Definitions of detectable and non-detectable antibody levels for each SARS-CoV-2 spike-protein assay

Parameter	Assay			
	Siemens Healthineers Diagnostics (COV2G) IgG	Siemens Healthineers Diagnostics (sCOVG) IgG	Roche Diagnostics Elecsys (Cov-2 spike)	DiaSorin (Trimeric S) IgG
Test usage[a]	February 2021 to November 2021	November 2021 to June 2022	March 2021 to June 2022	November 2021 to June 2022
Range	1 to 20 index units	1 to 150 index units	0.8 to 2,500 units/mL	13 to 800 AU/mL
Detectable antibody level	Result of ≥1 to ≤20 index units	Result of ≥1 to ≤100 index units (Centaur)<br>Result of ≥1 to ≤150 index units (Atellica)	Result of ≥0.8 to ≤2,500 units/mL	Result of ≥13 to ≤800 AU/mL
Non-detectable antibody level	Result of <1 index units	Result of <1 index units	Result of <0.8 units/mL	Result of <13 AU/mL

[a] The study data coverage spans from 1 July 2020 to 5 June 2022.

Abbreviation: SARS-CoV-2, severe acute respiratory syndrome coronavirus-2.

eTable 2. Outcome definitions

**Outcome of Interest**	**Definition**
SARS-CoV-2 infection	An inpatient or outpatient claim with an ICD-10 diagnosis code of U07.1 in any position.<br>*AND/OR*<br>A recorded positive SARS-CoV-2 diagnostic NAAT result.
Hospitalization with an associated SARS-CoV-2 infection	Any claim for an inpatient stay with an ICD-10 diagnosis code of U07.1 in any position.<br>*OR*<br>A recorded positive SARS-CoV-2 diagnostic NAAT test result during the inpatient stay or up to 14 days before inpatient admission.
All-cause mortality	Death due to any cause.
Any severe outcome	An outcome composite of hospitalization with an associated SARS-CoV-2 infection OR all-cause mortality.

Abbreviations: ICD-10, International Classification of Diseases, Tenth Revision; NAAT, n*ucleic acid amplification test;* SARS-CoV-2, severe acute respiratory syndrome coronavirus-2.

eTable 3. Comorbid conditions and other covariate definitions

Covariate	Definition
Year/season of Index	*Winter 2020-2021:* November 1, 2020 to February 28, 2021<br>*Spring 2021:* March 1, 2021 to May 30, 2021<br>*Summer 2021:* June 1, 2021 to August 30, 2021<br>*Fall 2021:* September 1, 2021 to November 30, 2021<br>*Winter 2021:* December 2021
Age	Recorded age on the index date
Sex	Recorded sex on the index date
Region	*Northeast*: Connecticut, Massachusetts, Vermont, Rhode Island, Maine, New York, New Jersey, New Hampshire, Pennsylvania<br>*Midwest*: Iowa, Illinois, Indiana, Kansas, Michigan, Minnesota, Missouri, North Dakota, Nebraska, Ohio, South Dakota, Wisconsin<br>*South*: Alabama, Arkansas, District of Columbia, Delaware, Florida, Georgia, Kentucky, Louisiana, Maryland, Mississippi, North Carolina, Oklahoma, South Carolina, Tennessee, Texas, Virginia, West Virginia<br>*West*: Alaska, Arizona, California, Colorado, Hawaii, Idaho, Montana, New Mexico, Nevada, Oregon, Utah, Washington, Wyoming<br>*Other/Missing/Unknown*: Armed Forces Americas, Armed Forces, Armed Forces Pacific, American Samoa, Micronesia, Guam, Marshall Islands, Northern Marianas Islands, Puerto Rico, Palau, Virgin Islands, (Missing)
SNF or nursing home utilization during the baseline period.	The occurrence of Medical Claims with the following attributes:<br>Place of Service (Standard) is any of: NURSING FACILITY, SKILLED NURSING FACILITY<br>OR<br>Revenue Code is any of: 0550, 0551, 0552, 0553, 0556, 0559, 0022<br>OR<br>Bill Type (Standard) is any of: 210, 211, 212, 213, 214, 215, 216, 217, 218, 219, 21A, 21B, 21C, 21D, 21E, 21F, 21G, 21H, 21I, 21J, 21K, 21M, 21N, 21O, 21P, 21Q, 21X, 21Y, 21Z, 220, 221, 222, 223, 224, 225, 226, 227, 228, 229, 22A, 22B, 22C, 22D, 22E, 22F, 22G, 22H, 22I, 22J, 22K, 22M, 22N, 22O, 22Q, 22X, 22Y, 22Z, 22`, 230, 231, 232, 233, 234, 235, 237, 238, 239, 23A, 23B, 23C, 23D, 23E, 23F, 23G, 23H, 23I, 23J, 23K, 23M, 23N, 23O, 23Q, 23X, 23Y, 23Z, 240, 241, 242, 243, 244, 245, 247, 248, 249, 24A, 24B, 24C, 24D, 24E, 24F, 24G, 24H, 24I, 24J, 24K, 24M, 24N, 24O, 24X, 24Y, 24Z, 250, 251, 252, 253, 254, 255, 257, 258, 259, 25A, 25B, 25C, 25D, 25E, 25F, 25G, 25H, 25I, 25J, 25K, 25M, 25N, 25O, 25X, 25Y, 25Z, 260, 261, 262, 263, 264, 265, 267, 268, 269, 26A, 26B, 26C, 26D, 26E, 26F, 26G, 26H, 26I, 26J, 26K, 26M, 26N, 26O, 26X, 26Y, 26Z, 270, 271, 272, 273, 274, 275, 277, 278, 279, 27A, 27B, 27C, 27D, 27E, 27F, 27G, 27H, 27I, 27J, 27K, 27M, 27N, 27O, 27X, 27Y, 27Z, 280, 281, 282, 283, 284, 285, 287, 288, 289, 28A, 28B, 28C, 28D, 28E, 28F, 28G, 28H, 28I, 28J, 28K, 28M, 28N, 28O, 28X, 28Y, 28Z<br>OR<br>Procedure Code, HCPCS and CPT is any of: 94004, 99307, 99308, 99310, 99313, 99315, 99316, 99304, 99305, 99306, 99309, 99311, 99312, 99318, 99301, 99302, 99303

At least one immunocompromising condition	Evidence of at least one of the following conditions: blood transplant, stem cell transplant, organ transplant with immunosuppressive therapy, cancer, primary immunodeficiency, HIV infection, immunosuppressive therapy, or other immunocompromising conditions.<br><br>eTable 4 contains detailed definitions of each of these conditions.
At least one vulnerable condition	Evidence of at least one of the following conditions: age ≥ 65 years, stage 4/5 chronic kidney disease or end-stage renal disease, chronic liver disease, chronic lung disease (moderate to severe asthma, bronchiectasis, bronchopulmonary dysplasia, COPD, as interstitial lung disease, pulmonary embolism, pulmonary hypertension), diabetes (type 1 or type 2), heart conditions (heart failure, coronary artery disease, cardiomyopathies, and hypertension), obesity, pregnancy, history of smoking/tobacco use, stroke or cerebrovascular disease, and tuberculosis.<br><br>eTable 4 contains detailed definitions of each of these conditions.
Vaccination status on the index date.	*COVID-19 fully vaccinated plus a booster:* Received 1 booster dose of either the Pfizer-BioNTech or Moderna COVID-19 vaccine at least 5 months after the final dose of the primary series of Pfizer/Moderna or at least 2 months after a Johnson & Johnson/Janssen COVID-19 vaccine.<br><br>*Fully vaccinated:* Considered fully vaccinated 14 days after meeting the following criteria: 2 doses of the Moderna or Pfizer-BioNTech COVID-19 vaccine given at least 21 days apart or 1 dose of Johnson & Johnson/Janssen COVID-19 vaccine.<br><br>*Partially vaccinated:* Considered partially vaccinated 14 days after meeting the following criteria:1 dose of the Pfizer-BioNTech COVID-19 vaccine or Moderna COVID-19 vaccine.<br><br>*Unvaccinated (ie, no evidence of a vaccination):* No evidence of receiving the Pfizer-BioNTech COVID-19 vaccine, the Moderna COVID-19 vaccine, or the Johnson & Johnson/Janssen COVID-19 vaccine.

Abbreviations: COPD, chronic obstructive pulmonary disease; COVID-19, coronavirus disease 2019; CPT, Current Procedural Terminology; HCPCS, Healthcare Common Procedure Coding System; HIV, Human immunodeficiency virus; SNF, skilled nursing facility.

eTable 4. List and definitions of immunocompromising and vulnerable conditions

Immunocompromising conditions	
**Condition**	**Definition**
Blood transplant	Blood transplant 2 years prior to index date.<br>Defined as a medical claim with at least one of the following ICD-10 diagnosis codes: T86.00, T86.09, Z48.290, Z94.81<br>OR<br>At a medical claim with at least one of the following ICD-10 procedure codes:<br>30230AZ, 30233AZ, 30240AZ, 30253Y1, 30260G1, 30260Y1, 30263G1, 30243AZ, 30250G1, 30250X1, 30250Y1, 30253G1, 30253X1, 30260X1, 30263X1, 30263Y1, 30230G2, 30230G3, 30230G4, 30230X1, 30230X3, 30230X4, 30230Y1, 30230Y2, 30230Y3, 30230Y4, 30233G1, 30233G4, 30233X1, 30233X2, 30233X3, 30233X4, 30233Y1, 30240G1, 30240G2, 30240G3, 30240G4, 30240X2, 30240X4, 30240Y2, 30240Y4, 30243G1, 30243G2, 30243G3, 30243X1, 30243X4, 30243Y2, 30243Y3, 30243Y4, 30230G1, 30230X2, 30233G2, 30233G3, 30233Y2, 30233Y3, 30233Y4, 30240X1, 30240X3, 30240Y1, 30240Y3, 30243G4, 30243X2, 30243X3, 30243Y1
Stem cell transplant	Stem cell transplant 2 years prior to index date.<br><br>Defined as evidence of a medical claim with at least one of the following ICD-10 procedure codes:<br>30230AZ, 30230G1, 30230G3, 30230G4, 30230X1, 30230X2, 30230Y1, 30230Y3, 30233G3, 30233G4, 30233X2, 30233X4, 30233Y1, 30233Y2, 30233Y4, 30240AZ, 30240G1, 30240G2, 30240G3, 30240G4, 30240X1, 30240X3, 30243G2, 30243G4, 30243X1, 30243X2, 30243X4, 30243Y1, 30243Y2, 30243Y4, 30250G1, 30253G1, 30253X1, 30253Y1, 30260G1, 30260Y1, 30263G1, 30263X1, 30230G2, 30230X3, 30230X4, 30230Y2, 30230Y4, 30233AZ, 30233G1, 30233G2, 30233X1, 30233X3, 30233Y3, 30240X2, 30240X4, 30240Y0, 30240Y1, 30240Y2, 30240Y3, 30240Y4, 30243AZ, 30243G1, 30243G3, 30243X3, 30243Y3, 30250X1, 30250Y1, 30260X1, 30263Y1<br>OR<br>A medical claim with any of the following HCPCS codes: 38240, 38242, 38243, S2150
Organ transplant with immunosuppressive therapy	Any history of an organ transplant and evidence of immunosuppressive therapy (list of immunosuppressive therapies below) in the 60 days prior to index date.<br><br>Organ transplant was defined having as any medical claim with at least one of the following ICD-10 diagnosis codes: D84.821, D84.9, T86.19, T86.20, T86.298, T86.39, T86.49, T86.818, T86.859, T86.898, T86.899, Z48.21, Z48.280, Z48.288, Z94.82, Z94.83, T86.10, T86.30, T86.40, T86.819, T86.858, Z48.22, Z48.23, Z48.24, Z94.0, Z94.1, Z94.2, Z94.3, Z94.4<br>OR<br>Any medical claim with at least one of the following ICD-10 procedure codes: 02YA0Z0, 02YA0Z2, 07YM0Z1, 07YM0Z2, 07YP0Z1, 0BYC0Z1, 0BYC0Z2, 0BYD0Z2, 0BYF0Z0, 0BYG0Z0, 0BYG0Z1, 0BYH0Z0, 0BYH0Z2, 0BYJ0Z1, 0BYJ0Z2, 0BYK0Z1, 0BYK0Z2, 0BYL0Z0, 0BYL0Z1, 0BYL0Z2, 0BYM0Z0, 0BYM0Z2, 0DY60Z0, 0DY60Z1, 0DY60Z2, 0DY80Z1, 0DY80Z2, 0DYE0Z0, 0DYE0Z1, 0DYE0Z2, 0FY00Z1, 0TY10Z0, 0TY10Z1, 3E1M39Z, 5A1D70Z, 5A1D90Z, BT2900Z, BT290ZZ, BT2910Z, BT29Y0Z, BT29ZZZ, BT39ZZZ, 02YA0Z1, 07YM0Z0, 07YP0Z0, 07YP0Z2, 0BYC0Z0, 0BYD0Z0, 0BYD0Z1, 0BYF0Z1, 0BYF0Z2, 0BYG0Z2, 0BYH0Z1, 0BYJ0Z0, 0BYK0Z0, 0BYM0Z1, 0DY50Z0, 0DY50Z1, 0DY50Z2, 0DY80Z0, 0FY00Z0, 0FY00Z2, 0FYG0Z0, 0FYG0Z1, 0FYG0Z2, 0TY00Z0, 0TY00Z1, 0TY00Z2, 0TY10Z2, 5A1D00Z, 5A1D80Z, BT291ZZ, BT29YZZ, BT39Y0Z, BT39YZZ, BT49ZZZ<br>OR<br>Any medical claim with at least one of the following HCPCS codes: 47135, 47136, 50365, A4673, A4674, A4680, A4700, A4705, A4708, A4712, A4714, A4719, A4720, A4723, A4724, A4730, A4736, A4740, A4750, A4765, A4850, A4870, A4900, A4901, A4905, A4912, A4913, A4914, A4918, E1500, E1530, E1575, E1590, E1592, E1594, E1610, E1615, E1620, E1632, E1635, S2053, S2054, S2060, S2065, S2142, 32851, 32852, 32853, 32854, 33935, 33945, 44135, 44136, 48554, 50360, 50370, A4653, A4671, A4672, A4690, A4706, A4707, A4709, A4721, A4722, A4725, A4726, A4728, A4735, A4737, A4755, A4760, A4766, A4802, A4820, A4860, A4880, A4890, A4910, A4911, E1510, E1520, E1540, E1550, E1560, E1570, E1580, E1600, E1625, E1630, E1634, E1636, S2152
Cancer	Active cancer therapy (defined as a therapy prescribed in the 180 days prior to index) and a cancer diagnosis in the 365 days prior to therapy.<br><br>Cancer malignancy was defined as having any medical claim with at least one of the following ICD-10 diagnosis codes: C00*, C01*, C02*, C03*, C04*, C05*, C06*, C07*, C08*, C09*, C10*, C11*, C12*, C13*, C14*, C15*, C16*, C17*, C18*, C19*, C20*, C21*, C22*, C23*, C24*, C25*, C26*, C30*, C31*, C32*, C33*,

	C34*, C37*, C38*, C39*, C40*, C41*, C43*, C45*, C46*, C47*, C48*, C49*, C50*, C51*, C52*, C53*, C54*, C55*, C56*, C57*, C58*, C60*, C61*, C62*, C63*, C64*, C65*, C66*, C67*, C68*, C69*, C70*, C71*, C72*, C73*, C74*, C75*, C76*, C81*, C82*, C83*, C84*, C85*, C88*, C90*, C91*, C92*, C93*, C94*, C95*, C96*, C97*<br><br>Use of cancer therapy was defined as having any medical claim or pharmacy claim with at least one of the following NDC codes:<br>00002298026, 00002298060, 00002397760, 00002416502, 00002416507, 00002416530, 00002416534, 00002416579, 00002418402, 00002418407, 00002418430, 00002448354, 00002481554, 00002533754, 00002621654, 00002719001, 00002750101, 00002750201, 00002762301, 00002764001, 00002766901, 00002767801, 00002771601, 00002892601, 00003029305, 00003029320, 00003029328, 00003031505, 00003031520, 00003049420, 00003052411, 00003052711, 00003052811, 00003083050, 00003085222, 00003085522, 00003085722, 00003229111, 00003232711, 00003232822, 00003373413, 00003377211, 00003377412, 00003452211, 00004015549, 00004015649, 00004016949, 00004023909, 00004024009, 00004024126, 00004024133, 00004024208, 00004035009, 00004035039, 00004035239, 00004035730, 00004036030, 00004036530, 00004110020, 00004110150, 00004110175, 00006007201, 00006007228, 00006007231, 00006007258, 00006007282, 00006046101, 00006046102, 00006046106, 00006046130, 00006046201, 00006046206, 00006046230, 00006046401, 00006046405, 00006046410, 00006056840, 00006302602, 00006302604, 00006302902, 00006306100, 00006306101, 00006306102, 00006306104, 00006306601, 00006306603, 00006386203, 00006388432, 00006394101, 00006394132, 00006404500, 00006404501, 00006404541, 00006410901, 00006410902, 00006410906, 00006410909, 00006411901, 00006411903, 00006412101, 00006412102, 00006503302, 00006503402, 00007326031, 00007326036, 00007326101, 00007326201, 00007420101, 00007420511, 00007420711, 00007440101, 00007440106, 00008010001, 00008117901, 00008451001, 00009000302, 00009000501, 00009001103, 00009001104, 00009001201, 00009001305, 00009001306, 00009001612, 00009001820, 00009002001, 00009002201, 00009003101, 00009003906, 00009003928, 00009003930, 00009003932, 00009003933, 00009004401, 00009004704, 00009004722, 00009004725, 00009004726, 00009004727, 00009004902, 00009005002, 00009005011, 00009005602, 00009005603, 00009005604, 00009005605, 00009006404, 00009006406, 00009007301, 00009011312, 00009017601, 00009019009, 00009027401, 00009028002, 00009028003, 00009028051, 00009028052, 00009028603, 00009030602, 00009030612, 00009062601, 00009069801, 00009069802, 00009075801, 00009076502, 00009079601, 00009082501, 00009090013, 00009090020, 00009090908, 00009090916, 00009091205, 00009092003, 00009111101, 00009111102, 00009307301, 00009307303, 00009307322, 00009307323, 00009338901, 00009347501, 00009347503, 00009347522, 00009347523, 00009509101, 00009509301, 00009752903, 00009752904, 00009752905, 00009766304, 00013013202, 00013111683, 00013113691, 00013114691, 00013115679, 00013117687, 00013126683, 00013128683, 00013220001, 00013220101, 00013220201, 00013252686, 00013257691, 00013258691, 00013259691, 00013871762, 00013872789, 00015050241, 00015050301, 00015050401, 00015050541, 00015050641, 00015059501, 00015059641, 00015191012, 00015191113, 00015301260, 00015303020, 00015303120, 00015303220, 00015308060, 00015321030, 00015321130, 00015321230, 00015321330, 00015321430, 00015321530, 00015321630, 00015335222, 00015335322, 00015340420, 00015355427, 00015355626, 00015356415, 00023590204, 00023590412, 00023590623, 00024022205, 00024059010, 00024059120, 00024060545, 00024061030, 00024065401, 00024065601, 00024079375, 00024515010, 00024515175, 00024582411, 00024584001, 00024584101, 00024584301, 00024584305, 00024586001, 00024586201, 00024591701, 00037122150, 00046110081, 00046110091, 00046110181, 00046110281, 00046110291, 00046110381, 00046110481, 00046110491, 00051002121, 00051002221, 00051002321, 00052060202, 00054001720, 00054001725, 00054001729, 00054001820, 00054001825, 00054001829, 00054001920, 00054001925, 00054006447, 00054008013, 00054014308, 00054014387, 00054016413, 00054024822, 00054024913, 00054026913, 00054027121, 00054027223, 00054032003, 00054032006, 00054032103, 00054032106, 00054032203, 00054032206, 00054032303, 00054032306, 00054032403, 00054032406, 00054032503, 00054038225, 00054038325, 00054039513, 00054039522, 00054039925, 00054048013, 00054048014, 00054048113, 00054048114, 00054049714, 00054317644, 00054317757, 00054317763, 00054372144, 00054372250, 00054372263, 00054412925, 00054413025, 00054417925, 00054418025, 00054418125, 00054418225, 00054418325, 00054418425, 00054418625, 00054449613, 00054449625, 00054449705, 00054449710, 00054449810, 00054449911, 00054455015, 00054455025, 00054458111, 00054458127, 00054460325, 00054460425, 00054472825, 00054472831, 00054474125, 00054474131, 00054474225, 00054817425, 00054817525, 00054817625, 00054817925, 00054818025, 00054818125, 00054818325, 00054849619, 00054855025, 00054860325, 00054860425, 00054872216, 00054872425, 00054873925, 00054874025, 00054981725, 00054981729, 00054982825, 00054982831, 00069006701, 00069007001, 00069007401, 00069007501, 00069007601, 00069007801, 00069007901, 00069008101, 00069008407, 00069008618, 00069009901, 00069010303, 00069010701, 00069010901, 00069013501, 00069013601, 00069014501, 00069014601, 00069014602, 00069014701, 00069014702, 00069014801, 00069014901, 00069015111, 00069015201, 00069015202, 00069015301, 00069015302, 00069015401, 00069015501, 00069017001, 00069017101, 00069017701, 00069017702, 00069017801, 00069017802, 00069017901, 00069017902, 00069018101, 00069018102, 00069018601, 00069018721, 00069018821, 00069018921, 00069019201, 00069019202, 00069019301, 00069019730, 00069020101, 00069020401, 00069020410, 00069020510, 00069020550, 00069022701, 00069023101, 00069023801, 00069024901, 00069028403, 00069029101, 00069029110, 00069029201, 00069029210, 00069029310, 00069029410, 00069029630, 00069029860, 00069030501, 00069030801, 00069031501, 00069032401,

	00069034201, 00069048603, 00069055038, 00069068803, 00069070012, 00069077038, 00069083038, 00069098038, 00069101001, 00069119530, 00069119830, 00069130510, 00069130610, 00069130710, 00069130810, 00069130904, 00069131110, 00069131810, 00069134002, 00069134005, 00069134016, 00069153130, 00069229930, 00069303020, 00069303120, 00069303220, 00069303320, 00069303420, 00069385710, 00069385810, 00069385910, 00069400405, 00069401510, 00069402625, 00069403001, 00069403101, 00069403201, 00069403301, 00069403401, 00069403701, 00069449522, 00069449622, 00069454101, 00069454102, 00069454301, 00069454302, 00069454501, 00069454502, 00069454701, 00069454702, 00069814020, 00069814120, 00069914111, 00069914122, 00069914211, 00069914222, 00069914411, 00069932122, 00074054130, 00074056111, 00074056114, 00074056607, 00074056611, 00074057611, 00074057622, 00074057634, 00074057928, 00074310832, 00074310932, 00074334603, 00074347303, 00074364203, 00074368303, 00074646332, 00074647932, 00074726950, 00075800120, 00075800180, 00075800301, 00075800404, 00078001705, 00078001715, 00078010205, 00078010215, 00078010901, 00078010961, 00078011022, 00078018001, 00078018061, 00078018101, 00078018161, 00078018201, 00078018261, 00078018325, 00078018425, 00078024015, 00078024061, 00078024115, 00078024161, 00078024615, 00078024661, 00078024815, 00078024861, 00078024915, 00078027422, 00078034061, 00078034161, 00078034261, 00078038725, 00078040134, 00078043815, 00078049561, 00078052651, 00078052687, 00078056651, 00078056661, 00078056751, 00078056761, 00078059061, 00078059251, 00078059287, 00078059451, 00078059461, 00078062051, 00078062061, 00078062651, 00078062661, 00078062751, 00078062761, 00078062851, 00078062861, 00078064070, 00078064515, 00078064681, 00078064781, 00078064881, 00078064930, 00078065006, 00078065106, 00078065206, 00078066615, 00078066815, 00078066913, 00078066961, 00078067066, 00078067119, 00078067201, 00078067301, 00078067461, 00078067515, 00078067615, 00078067919, 00078068019, 00078068166, 00078068266, 00078068306, 00078068361, 00078069061, 00078069484, 00078069802, 00078069819, 00078069851, 00078069899, 00078070184, 00078070802, 00078070891, 00078070956, 00078071502, 00078071591, 00078071656, 00078081181, 00078081881, 00078082581, 00078084619, 00078086001, 00078086714, 00078086742, 00078087421, 00078087463, 00078090961, 00078091661, 00078092361, 00078095819, 00085053901, 00085057102, 00085111001, 00085113301, 00085116801, 00085123501, 00085124201, 00085125401, 00085128702, 00085128703, 00085131201, 00085131202, 00085136601, 00085136602, 00085136603, 00085136604, 00085136605, 00085138101, 00085138801, 00085138802, 00085141701, 00085141702, 00085141703, 00085142501, 00085142502, 00085142503, 00085142504, 00085142505, 00085143001, 00085143002, 00085143003, 00085143004, 00085143005, 00085151901, 00085151902, 00085151903, 00085151904, 00085151905, 00085300401, 00085300402, 00085300403, 00085300404, 00085300405, 00085434701, 00085434801, 00085434901, 00085435001, 00085435101, 00085435201, 00088111114, 00088120205, 00088120243, 00088120305, 00088120329, 00088120343, 00088120632, 00088120806, 00088120876, 00088120926, 00089061012, 00093022001, 00093022056, 00093023319, 00093023333, 00093023356, 00093023393, 00093078201, 00093078205, 00093078210, 00093078256, 00093078405, 00093078406, 00093078410, 00093078486, 00093112589, 00093551006, 00093565556, 00093565598, 00093574019, 00093574065, 00093574119, 00093574165, 00093574219, 00093574265, 00093611816, 00093611887, 00093612619, 00093612664, 00093723619, 00093723633, 00093723656, 00093723693, 00093729001, 00093729010, 00093729056, 00093730119, 00093730165, 00093730203, 00093730219, 00093730265, 00093735501, 00093735505, 00093735556, 00093735598, 00093747306, 00093747489, 00093748512, 00093748519, 00093748520, 00093753656, 00093759941, 00093759957, 00093760041, 00093760057, 00093760141, 00093760157, 00093760257, 00093762056, 00093762998, 00093763056, 00093763841, 00093763857, 00093763941, 00093763957, 00093766356, 00093766456, 00093776624, 00093776724, 00093776824, 00093901865, 00093901965, 00093902065, 00095008735, 00095008851, 00095008921, 00115140801, 00115143808, 00115143810, 00115147623, 00115147659, 00115167572, 00115167573, 00115169606, 00115169701, 00115170001, 00115703701, 00121075908, 00121077308, 00121077708, 00121090204, 00143120201, 00143125401, 00143125425, 00143142501, 00143147301, 00143147310, 00143147325, 00143147501, 00143147510, 00143147525, 00143147701, 00143147705, 00143147710, 00143147725, 00143242230, 00143242330, 00143242407, 00143920201, 00143920301, 00143920401, 00143921701, 00143921801, 00143921901, 00143924001, 00143924101, 00143927001, 00143927501, 00143927701, 00143929001, 00143929101, 00143930601, 00143930701, 00143930801, 00143938401, 00143953101, 00143954601, 00143954701, 00143954801, 00143954810, 00143954901, 00143954910, 00143955001, 00143955110, 00143955201, 00143955301, 00143955401, 00143955501, 00143955801, 00143956501, 00143958301, 00143959721, 00143960601, 00143964201, 00143970001, 00143970101, 00143970201, 00143973801, 00143973805, 00143973810, 00143973901, 00143973910, 00143974001, 00143974010, 00143974401, 00143974410, 00143974501, 00143974505, 00143975001, 00143977106, 00143983001, 00143989001, 00143989010, 00143989101, 00143989105, 00143989125, 00172496058, 00172496070, 00172524060, 00172524160, 00172731000, 00172731046, 00172731100, 00172731146, 00172731200, 00172731246, 00172731320, 00173013093, 00173044200, 00173044202, 00173044600, 00173044602, 00173044604, 00173044700, 00173044702, 00173044704, 00173048900, 00173056900, 00173057000, 00173057004, 00173063535, 00173071204, 00173071215, 00173071225, 00173071325, 00173075200, 00173080409, 00173080802, 00173080805, 00173082101, 00173082102, 00173082133, 00173084608, 00173084708, 00173084813, 00173084913, 00173089601, 00178058201, 00178058208, 00179009944, 00179010044, 00182186389, 00182186400, 00182186489, 00185015501, 00185015601, 00185093230, 00185093287, 00185093330, 00185093386, 00185093387, 00185740014, 00185740085, 00187090101, 00187090201, 00187122103, 00187320447, 00187552560, 00187552675, 00245057501, 00310020130, 00310020137, 00310048230, 00310061060,

	00310062528, 00310062560, 00310065758, 00310066812, 00310066860, 00310067912, 00310067960, 00310070510, 00310070530, 00310070539, 00310072010, 00310072025, 00310072050, 00310095036, 00310095130, 00310134930, 00310135030, 00310450012, 00310461150, 00310470001, 00310471511, 00310772010, 00310781030, 00310782030, 00310783030, 00310784030, 00338000801, 00338006301, 00338006701, 00338008001, 00338008601, 00338130501, 00338130503, 00338176241, 00338399101, 00338399301, 00378001401, 00378014405, 00378014491, 00378027401, 00378027493, 00378031553, 00378031593, 00378034453, 00378034493, 00378064001, 00378064010, 00378064101, 00378064110, 00378064201, 00378064205, 00378064210, 00378100394, 00378145201, 00378145205, 00378145401, 00378145405, 00378145801, 00378145805, 00378145877, 00378204201, 00378204293, 00378207105, 00378207193, 00378224577, 00378224693, 00378251191, 00378251278, 00378309685, 00378309785, 00378309885, 00378315101, 00378315177, 00378315193, 00378326694, 00378354725, 00378354752, 00378471022, 00378471522, 00378473022, 00378479106, 00378500193, 00378503677, 00378503693, 00378526014, 00378526098, 00378526114, 00378526198, 00378526214, 00378526298, 00378526314, 00378526398, 00378526414, 00378526498, 00378526598, 00378603405, 00378603477, 00378603493, 00378661185, 00378661193, 00378661285, 00378661293, 00378661485, 00378661488, 00378661493, 00378686801, 00378686901, 00378692078, 00378692191, 00378695501, 00378701705, 00378701793, 00378709601, 00378709693, 00378713193, 00378713293, 00378713393, 00378773293, 00378773493, 00378773497, 00378800977, 00378800993, 00378817091, 00378817191, 00378817291, 00378904001, 00378904005, 00378904501, 00378904505, 00409018101, 00409018125, 00409018201, 00409018225, 00409018301, 00409018325, 00409018501, 00409018601, 00409018701, 00409020102, 00409020110, 00409020120, 00409020125, 00409020126, 00409020127, 00409030201, 00409030225, 00409032320, 00409036601, 00409036701, 00409036801, 00409080101, 00409080109, 00409111201, 00409112011, 00409112012, 00409112062, 00409250410, 00409321705, 00409321805, 00409341401, 00409421501, 00409421505, 00409422901, 00409475501, 00409475502, 00409475503, 00409475512, 00409475518, 00409475901, 00409476013, 00409485605, 00409568401, 00409568423, 00409568502, 00409568523, 00430072024, 00430072124, 00430072224, 00440816512, 00440816710, 00440816715, 00469012599, 00469062599, 00469072560, 00469142590, 00517042001, 00517044001, 00517074501, 00517074601, 00517092008, 00517490125, 00517490525, 00517493025, 00527145006, 00527145106, 00527145206, 00527293037, 00527293043, 00527293137, 00527293237, 00527293243, 00527293337, 00527293341, 00527293437, 00527293441, 00527293537, 00555030102, 00555030138, 00555044605, 00555044609, 00555044663, 00555048401, 00555048402, 00555048527, 00555057202, 00555057235, 00555060602, 00555060702, 00555060703, 00555060704, 00555077902, 00555077904, 00555080802, 00555087202, 00555087204, 00555087302, 00555087304, 00555088202, 00555088602, 00555088604, 00555088702, 00555088704, 00555089902, 00555090401, 00555090405, 00555090414, 00555105456, 00555105486, 00555105556, 00555105586, 00555105686, 00555105756, 00555105786, 00574010601, 00574010603, 00574014804, 00574079201, 00574082105, 00574086610, 00574087005, 00574087205, 00591048701, 00591048705, 00591048801, 00591048805, 00591052801, 00591079001, 00591079021, 00591222215, 00591222315, 00591222455, 00591223218, 00591223260, 00591223319, 00591223330, 00591229219, 00591229230, 00591236701, 00591236710, 00591236730, 00591243315, 00591243415, 00591243515, 00591243615, 00591245115, 00591246618, 00591247218, 00591247260, 00591247319, 00591247330, 00591250115, 00591289749, 00591322126, 00591356279, 00591356355, 00591359160, 00591359260, 00591359360, 00591413054, 00591438579, 00591501902, 00591505201, 00591505210, 00591505221, 00591505243, 00591544201, 00591544205, 00591544210, 00591544221, 00591544243, 00591544301, 00591544305, 00591544310, 00597013730, 00597013830, 00597014130, 00597014360, 00597014560, 00603114756, 00603156756, 00603156758, 00603363302, 00603363316, 00603363321, 00603363328, 00603389919, 00603390021, 00603390121, 00603418016, 00603459315, 00603459321, 00603533521, 00603533532, 00603533621, 00603533715, 00603533721, 00603533731, 00603533732, 00603533815, 00603533821, 00603533828, 00603533831, 00603533832, 00603533921, 00603533928, 00603533932, 00603938856, 00641036721, 00641036725, 00641607801, 00641607825, 00641607901, 00641608001, 00641608025, 00641614501, 00641614525, 00641614601, 00641614625, 00641617401, 00641617410, 00641617501, 00641617510, 00641617601, 00641617610, 00641617701, 00641617801, 00703003101, 00703003104, 00703004301, 00703004501, 00703005101, 00703005104, 00703006301, 00703024101, 00703024301, 00703024501, 00703301513, 00703301812, 00703301912, 00703306711, 00703306911, 00703312508, 00703315401, 00703315501, 00703321301, 00703321381, 00703321601, 00703321681, 00703321701, 00703321801, 00703321881, 00703324911, 00703330101, 00703330104, 00703331101, 00703331104, 00703332101, 00703332104, 00703332194, 00703333301, 00703334301, 00703342711, 00703342911, 00703352401, 00703352403, 00703367101, 00703367103, 00703367191, 00703367193, 00703367301, 00703367501, 00703367591, 00703367801, 00703367881, 00703398501, 00703398601, 00703400401, 00703401411, 00703401418, 00703408551, 00703409401, 00703410048, 00703410058, 00703410068, 00703415411, 00703415491, 00703415511, 00703415591, 00703415611, 00703415691, 00703418201, 00703418291, 00703418301, 00703418391, 00703423901, 00703423981, 00703424401, 00703424481, 00703424601, 00703424681, 00703424801, 00703424881, 00703424891, 00703440211, 00703441211, 00703443211, 00703443281, 00703443411, 00703443481, 00703450204, 00703450284, 00703450294, 00703463601, 00703468001, 00703468501, 00703468601, 00703471401, 00703471471, 00703476401, 00703476481, 00703476601, 00703476681, 00703476701, 00703476801, 00703476881, 00703480501, 00703480503, 00703485211, 00703485291, 00703504001, 00703504301, 00703504303, 00703504601, 00703507501, 00703507503, 00703514001, 00703514501, 00703514591, 00703523311, 00703523313, 00703523391, 00703523393, 00703565301, 00703565601, 00703565691, 00703565701,

	00703565791, 00703572001, 00703573001, 00703574711, 00703574811, 00703577501, 00703577801, 00703585401, 00703722101, 00703722102, 00703722103, 00703722104, 00703722601, 00703722603, 00703723939, 00703787103, 00703789101, 00703797101, 00703797103, 00703797301, 00781167931, 00781167933, 00781168131, 00781168133, 00781211901, 00781211931, 00781232106, 00781232151, 00781232246, 00781232268, 00781232368, 00781269144, 00781269175, 00781269244, 00781269275, 00781269344, 00781269375, 00781269444, 00781269475, 00781269544, 00781269575, 00781269675, 00781301072, 00781301095, 00781301180, 00781302975, 00781303075, 00781303175, 00781307912, 00781308475, 00781313171, 00781313195, 00781313271, 00781313295, 00781313670, 00781313775, 00781313980, 00781316475, 00781316575, 00781320194, 00781323394, 00781324375, 00781324494, 00781324572, 00781325394, 00781325594, 00781328275, 00781328379, 00781329680, 00781331275, 00781331570, 00781331780, 00781341575, 00781349212, 00781349775, 00781400332, 00781406336, 00781502201, 00781502207, 00781523806, 00781523864, 00781523906, 00781523964, 00781523980, 00781532501, 00781532531, 00781535631, 00781540901, 00781540931, 00781540964, 00781715209, 00781916475, 00781916575, 00781925394, 00781931570, 00781931780, 00832008600, 00832028500, 00832059530, 00904267460, 00904357161, 00904601260, 00904601946, 00904619546, 00904620846, 00904620946, 00904622961, 00904655161, 00904655261, 00904657461, 00904674561, 00904674604, 00904691461, 00904694804, 00944262001, 00944262002, 00944262003, 00944262004, 00944265503, 00944265504, 00944265603, 00944265804, 00944381001, 00955102001, 00955102104, 00955102208, 00955172510, 00955172720, 00955173110, 00955173320, 00955174601, 10019090501, 10019090502, 10019090503, 10019090517, 10019090603, 10019090604, 10019090605, 10019090663, 10019092501, 10019092602, 10019093501, 10019093601, 10019093701, 10019093801, 10019093901, 10019094201, 10019094301, 10019094401, 10019094501, 10019095301, 10019095302, 10019095362, 10019095501, 10019095601, 10019095701, 10019098201, 10019098401, 10139006202, 10139006210, 10139006240, 10139006301, 10139006310, 10139006311, 10139006312, 10139006320, 10139006350, 10370026801, 10544021206, 10544047320, 10544053830, 10544057506, 10544091330, 10544091420, 10544091421, 10544091510, 10544091530, 10631000231, 10631000331, 10631000531, 10631000731, 10631011531, 10631011631, 10631011731, 10631011831, 10631013331, 10631013431, 10768708501, 10768728303, 10768728304, 10768773303, 10768773304, 10885000101, 11399000501, 11399000530, 11994001601, 13632012301, 13668045301, 13668046201, 13668059181, 13668059182, 13668059284, 13668059286, 13668059386, 13668059487, 13925016604, 13925050104, 13925052301, 14789060010, 15014021121, 15054004301, 15054006001, 15054009001, 15054012001, 15054106003, 15054106004, 15054109003, 15054109004, 15054112003, 15054112004, 16477050501, 16477050521, 16477050548, 16477051008, 16571042103, 16590014921, 16590026910, 16590032610, 16590032615, 16590032620, 16590032621, 16590032630, 16590036521, 16590037321, 16590037330, 16590062421, 16590062448, 16714000101, 16714002701, 16714008801, 16714008825, 16714008901, 16714009001, 16714011802, 16714012001, 16714013001, 16714013025, 16714013101, 16714013701, 16714014001, 16714015001, 16714022101, 16714022110, 16714022112, 16714022130, 16714022132, 16714046501, 16714046701, 16714046801, 16714047201, 16714047301, 16714050001, 16714052201, 16714052203, 16714052204, 16714052205, 16714052210, 16714057101, 16714057102, 16714067101, 16714067102, 16714072501, 16714072601, 16714072701, 16714072801, 16714074201, 16714077701, 16714081501, 16714081601, 16714081602, 16714083401, 16714085601, 16714085701, 16714085801, 16714085901, 16714088601, 16714089001, 16714090801, 16714090901, 16714091501, 16714092701, 16714092801, 16714092901, 16714093001, 16714096301, 16729002301, 16729002310, 16729003410, 16729003415, 16729003510, 16729003515, 16729003516, 16729004853, 16729004854, 16729004953, 16729004954, 16729005053, 16729005054, 16729005153, 16729007212, 16729007329, 16729009001, 16729009010, 16729009015, 16729009016, 16729009203, 16729010811, 16729011408, 16729011411, 16729011431, 16729011505, 16729011638, 16729011711, 16729011838, 16729012049, 16729012953, 16729012954, 16729013053, 16729013054, 16729015131, 16729022361, 16729022405, 16729022850, 16729023163, 16729023164, 16729023165, 16729024003, 16729024330, 16729024331, 16729024605, 16729024711, 16729024838, 16729026231, 16729026763, 16729026764, 16729026765, 16729027603, 16729027605, 16729027611, 16729027638, 16729027667, 16729027668, 16729027703, 16729027730, 16729027735, 16729028811, 16729028838, 16729029512, 16729029531, 16729029533, 16729029534, 16729029783, 16729029805, 16729030610, 16729033203, 16729033205, 16729035192, 16729036566, 16729039130, 16729041903, 16729042333, 16729042605, 16729043630, 16729048601, 17156052401, 17478032705, 17478032745, 17478054601, 17478054602, 17478054604, 17478054605, 17478054701, 17478076106, 17478076206, 17478076306, 17856069105, 17856075902, 17856075905, 17856372201, 20482033530, 20536032201, 21695008021, 21695011100, 21695011130, 21695029030, 21695030530, 21695030620, 21695030621, 21695030628, 21695030630, 21695030636, 21695030639, 21695030642, 21695030650, 21695030710, 21695030712, 21695030714, 21695030715, 21695030720, 21695030721, 21695030730, 21695036001, 21695036010, 21695036508, 21695036516, 21695038030, 21695038204, 21695038208, 21695038220, 21695038260, 21695058005, 21695058014, 21695061300, 21695061330, 21695062300, 21695062330, 21695072812, 21695074512, 21695076421, 21695076521, 21695076548, 21695082940, 21695083404, 21695083412, 21695083415, 21695083430, 21695083530, 21695084910, 21695085005, 21695089610, 21695089620, 21695089630, 21695095205, 21695099030, 23155016831, 23155017031, 23155017931, 23155017932, 23155019631, 23155019642, 23155019643, 23155021331, 23155021431, 23155024041, 23155026141, 23155037731, 23155037831, 23155037841, 23155037842, 23155048331, 23155048431, 23155052831, 23155052931, 23155054731, 23155054741, 23155054742, 23155054831, 23155054841, 23155054842, 23155054931, 23155055031, 23155064941, 23155068531, 23155068631, 23594050501, 23594050502,

	23594050521, 23594050548, 23594050550, 23594051008, 23594091508, 24201010104, 24201023701, 24338005008, 24987011114, 25021020110, 25021020111, 25021020166, 25021020167, 25021020168, 25021020169, 25021020205, 25021020215, 25021020245, 25021020251, 25021020325, 25021020351, 25021020401, 25021020405, 25021020505, 25021020606, 25021020661, 25021020705, 25021020725, 25021020751, 25021020810, 25021020950, 25021021120, 25021021250, 25021021305, 25021021317, 25021021350, 25021021402, 25021021405, 25021021598, 25021021599, 25021022160, 25021022201, 25021022204, 25021022207, 25021023002, 25021023005, 25021023120, 25021023310, 25021023320, 25021023410, 25021023550, 25021023604, 25021023706, 25021023905, 25021023926, 25021023952, 25021024110, 25021024202, 25021024501, 25021024504, 25021024602, 25021045101, 25021045201, 25021045301, 25021045405, 25021045505, 25021046274, 25021077702, 25021077801, 25021077866, 25021077901, 25021078104, 25021078220, 25021078305, 25021078874, 25021080166, 25021080167, 25021080210, 25021080310, 25021080705, 25021080810, 25021081030, 25021081310, 25021081366, 25021081430, 25021081467, 25021081530, 25021081567, 25021081630, 25021081667, 25021082406, 25021082666, 25021082667, 25021082682, 25021082850, 29336061012, 29336061024, 30237890006, 30698001701, 30698001730, 30698010230, 30698012005, 30698012105, 30698020101, 30698020130, 30698020201, 30698020230, 31722013130, 31722013190, 31722052501, 31722052510, 31722052530, 31722052590, 31722096060, 31722096160, 31722096260, 35356019730, 35356021030, 35356024900, 35356025000, 35356025100, 35356042630, 35356044530, 35356065210, 35356065220, 35356067320, 35356067321, 35356067330, 35356067415, 35356067418, 35356067420, 35356067430, 35356067715, 35356067720, 35356067721, 35356067728, 35356067730, 35356067740, 35356067760, 35356067790, 35356067830, 35356067910, 35356067930, 35356075565, 35356076321, 35356081810, 35356081815, 35356081818, 35356081820, 35356081821, 35356081830, 35356081915, 35356081920, 35356081921, 35356081930, 35356081940, 35356081942, 35356085306, 35356085309, 35356085310, 35356085320, 35356085330, 36000001225, 36000001301, 36000001406, 38423011001, 39822210002, 39822212001, 39822218001, 39822220001, 41616048583, 41616048588, 41616093640, 42023011001, 42023011101, 42023011201, 42023013401, 42023013501, 42023013601, 42023014901, 42023015101, 42043018003, 42043039000, 42043039002, 42043039020, 42043039021, 42043039040, 42195012106, 42195012707, 42195014912, 42195015021, 42195015049, 42195015110, 42195022106, 42195072121, 42238011101, 42238011112, 42254007710, 42254007730, 42254009710, 42254010208, 42254011030, 42254016001, 42254016130, 42254021210, 42254021310, 42254024330, 42291002412, 42291008530, 42291008590, 42291010530, 42291016660, 42291016712, 42291016830, 42291016850, 42291019060, 42291019112, 42291025790, 42291028030, 42291028050, 42291028090, 42291032101, 42291035190, 42291035230, 42291037490, 42291044960, 42291045060, 42291045160, 42291050501, 42291059401, 42291072610, 42291072690, 42291072710, 42291076801, 42291076901, 42291077050, 42291077101, 42291077150, 42291084301, 42292000701, 42292000710, 42292005105, 42292005205, 42292005305, 42292005703, 42367012121, 42367012125, 42367012129, 42367052025, 42367052125, 42388001114, 42388001214, 42388001314, 42388002326, 42388002426, 42388002526, 42543014050, 42543014101, 42543014201, 42549052221, 42549064714, 42549065730, 42549065760, 42549065790, 42658001001, 42658002101, 42747032730, 42747072601, 42747076101, 42799081201, 42799081301, 42806008701, 42806008705, 42806008801, 42806008805, 42806008901, 42806008905, 42806035801, 42806035830, 42806035925, 42806040001, 42806040021, 42858086706, 42858086806, 42858086906, 43063005202, 43063005204, 43063009706, 43063010910, 43063020101, 43063020130, 43063020190, 43063020801, 43063020830, 43063020860, 43063026607, 43063027304, 43063038306, 43063038630, 43063041501, 43063041530, 43063042610, 43063042612, 43063042620, 43063042621, 43063042628, 43063042630, 43063042640, 43063042642, 43063042650, 43063042660, 43063043210, 43063043212, 43063043215, 43063043220, 43063043221, 43063043230, 43063043805, 43063043810, 43063043890, 43063043930, 43063044601, 43063044630, 43063044690, 43063047225, 43063056002, 43063056003, 43063056004, 43063056005, 43063056006, 43063056010, 43063056020, 43063059005, 43063059006, 43063059009, 43063059010, 43063059012, 43063059014, 43063059015, 43063059018, 43063059020, 43063059021, 43063059025, 43063059030, 43063059210, 43063061021, 43063064321, 43063064355, 43063064410, 43063064415, 43063064420, 43063064421, 43063064430, 43063064440, 43063064442, 43063064450, 43063070306, 43063070309, 43063070310, 43063070315, 43063070318, 43063070320, 43063070321, 43063070325, 43063070330, 43063070630, 43063070690, 43063071730, 43063071790, 43063074612, 43063077006, 43063079206, 43066000101, 43066000601, 43066001001, 43066001401, 43066001801, 43288010410, 43288010810, 43353025360, 43353065760, 43353068760, 43353068860, 43353081960, 43547025801, 43598025811, 43598025940, 43598026202, 43598028335, 43598030330, 43598030390, 43598030562, 43598033011, 43598034431, 43598034490, 43598034530, 43598034531, 43598034837, 43598035804, 43598038957, 43598039248, 43598042737, 43598046562, 43598050501, 43598050510, 43598050530, 43598054125, 43598061040, 43598061111, 43598065011, 43598067811, 43598068235, 43598068325, 43598077111, 43598077311, 43598085911, 43598086560, 43598094811, 43975025205, 43975025214, 43975025305, 43975025314, 43975025405, 43975025414, 43975025505, 43975025514, 43975025605, 43975025614, 43975025705, 43975030710, 43975030810, 43975031510, 44087353501, 44087400000, 44087400004, 44087400005, 44087400006, 44087400007, 44087400008, 44087400009, 44087500003, 44087500006, 44206045824, 44523018208, 44567050501, 44567050601, 44567050701, 44567050901, 44567051001, 44567051101, 44567053001, 45629008901, 45629013401, 45802007662, 45802030321, 45802030367, 45802036853, 45802036862, 45802073321, 45802073367, 45963044055, 45963050002, 45963050008, 45963050030, 45963053830, 45963053930, 45963060755, 45963060756, 45963060860, 45963060868, 45963060955, 45963061153, 45963061159, 45963061257, 45963061353, 45963061356,

	45963061359, 45963061383, 45963061386, 45963061389, 45963061451, 45963061455, 45963061481, 45963061485, 45963061556, 45963061959, 45963062060, 45963062151, 45963062357, 45963062458, 45963063660, 45963063749, 45963063858, 45963064057, 45963064077, 45963068602, 45963073355, 45963073357, 45963073360, 45963073368, 45963073452, 45963073454, 45963073474, 45963076257, 45963076552, 45963079056, 46026098301, 47335003540, 47335004640, 47335004740, 47335004940, 47335005040, 47335008250, 47335008350, 47335015040, 47335015140, 47335015340, 47335015440, 47335017640, 47335017840, 47335028440, 47335028541, 47335032340, 47335040181, 47335047281, 47335047583, 47335048583, 47335048588, 47335058140, 47335058142, 47335071481, 47335071483, 47335071513, 47335071581, 47335071583, 47335089021, 47335089072, 47335089074, 47335089080, 47335089121, 47335089172, 47335089174, 47335089180, 47335089221, 47335089272, 47335089274, 47335089280, 47335089374, 47335089380, 47335089540, 47335092921, 47335092972, 47335092974, 47335092980, 47335093021, 47335093072, 47335093074, 47335093080, 47335093640, 47335096241, 47426010106, 47426020101, 47781010830, 47781020050, 47781057807, 47781059122, 47781059229, 47781059307, 47781059407, 47781059507, 47781060320, 47781060427, 47781060594, 47781060694, 47781060925, 47781061023, 47781062222, 47781062291, 48102004501, 48102004601, 48102004701, 48102004720, 48102004801, 48102005101, 48818000101, 48818000102, 49281088003, 49663000106, 49663000225, 49884008401, 49884008501, 49884008601, 49884008701, 49884011991, 49884012591, 49884012791, 49884012901, 49884028901, 49884029001, 49884029004, 49884029005, 49884032462, 49884032562, 49884036826, 49884037301, 49884072401, 49884075305, 49884075313, 49884086702, 49884086802, 49884086902, 49884092202, 49884092204, 49999000800, 49999000820, 49999000821, 49999000830, 49999000840, 49999000855, 49999002812, 49999002814, 49999002815, 49999002820, 49999002821, 49999002830, 49999002840, 49999002848, 49999002860, 49999002865, 49999005906, 49999005930, 49999008330, 49999010900, 49999010930, 49999010990, 49999011000, 49999011006, 49999011007, 49999011010, 49999011015, 49999011018, 49999011020, 49999011021, 49999011030, 49999015321, 49999015330, 49999045830, 50090008804, 50090008900, 50090009200, 50090010200, 50090016600, 50090016602, 50090016705, 50090029409, 50090029501, 50090043900, 50090049002, 50090049101, 50090065500, 50090065501, 50090094200, 50090095500, 50090100100, 50090101500, 5009010<br>OR<br>A pharmacy claim with at least one of the following cancer therapy generic names: ABARELIX, ABEMACICLIB, ABIRATERONE ACETATE, ACALABRUTINIB, ADO-TRASTUZUMAB EMTANSINE, ALDESLEUKIN, ALEMTUZUMAB, ALITRETINOIN, ALPELISIB, ALTRETAMINE, ANASTROZOLE, APALUTAMIDE, APREPITANT, ARSENIC TRIOXIDE, ASPARAGINASE, ATEZOLIZUMAB, AVAPRITINIB, AVELUMAB, AXICABTAGENE CILOLEUCEL, AXITINIB, AZACITIDINE, BELANTAMAB MAFODOTIN-BLMF, BELINOSTAT, BENDAMUSTINE HCL, BEVACIZUMAB, BEVACIZUMAB-AWWB, BEVACIZUMAB-BVZR, BEXAROTENE, BICALUTAMIDE, BINIMETINIB, BLEOMYCIN SULFATE, BLINATUMOMAB, BORTEZOMIB, BOSUTINIB, BRENTUXIMAB VEDOTIN, BREXUCABTAGENE AUTOLEUCEL, BRIGATINIB, BROMOCRIPTINE MESYLATE, BUSULFAN, CABAZITAXEL, CALASPARGASE PEGOL-MKNL, CAPECITABINE, CARBOPLATIN, CARFILZOMIB, CARMUSTINE, CEMIPLIMAB-RWLC, CERITINIB, CETUXIMAB, CHLORAMBUCIL, CISPLATIN, CLADRIBINE, CLOFARABINE, CORTISONE ACETATE, CRIZOTINIB, CYCLOPHOSPHAMIDE, CYCLOSPORINE, CYSTEAMINE BITARTRATE, CYTARABINE, DACARBAZINE, DACOMITINIB, DACTINOMYCIN, DARATUMUMAB, DAROLUTAMIDE, DASATINIB, DECITABINE, DENILEUKIN DIFTITOX, DENOSUMAB, DEXAMETHASONE, DEXAMETHASONE ACETATE, DEXAMETHASONE SODIUM PHOSPHATE, DIETHYLSTILBESTROL, DINUTUXIMAB, DOCETAXEL, DOLASETRON MESYLATE, DRONABINOL, DURVALUMAB, DUTASTERIDE, DUVELISIB, ELOTUZUMAB, ENASIDENIB MESYLATE, ENCORAFENIB, ENFORTUMAB VEDOTIN-EJFV, ENTRECTINIB, ENZALUTAMIDE, EPOETIN ALFA, EPOETIN ALFA-EPBX, ERDAFITINIB, ERIBULIN MESYLATE, ESTRADIOL, ESTRADIOL VALERATE, ESTRAMUSTINE PHOSPHATE SODIUM, ESTROGENS, CONJUGATED, ETIDRONATE DISODIUM, ETOPOSIDE, ETOPOSIDE PHOSPHATE, EVEROLIMUS, EXEMESTANE, FAM-TRASTUZUMAB DERUXTECAN-NXKI, FILGRASTIM, FILGRASTIM-AAFI, FILGRASTIM-SNDZ, FINASTERIDE, FLOXURIDINE, FLUDARABINE PHOSPHATE, FLUOROURACIL, FLUOXYMESTERONE, FLUTAMIDE, FOSAPREPITANT DIMEGLUMINE, FULVESTRANT, GEFITINIB, GEMCITABINE HCL, GEMTUZUMAB OZOGAMICIN, GLUCARPIDASE, GOSERELIN ACETATE, GRANISETRON, HISTRELIN ACETATE, HYDROCORTISONE, HYDROXYPROGESTERONE CAPROATE, HYDROXYUREA, IBRUTINIB, IDELALISIB, IFOSFAMIDE, IMATINIB MESYLATE, IMIQUIMOD, INOTUZUMAB OZOGAMICIN, IPILIMUMAB, ISOTRETINOIN, IVOSIDENIB, IXABEPILONE, KETOCONAZOLE, LANREOTIDE ACETATE, LENALIDOMIDE, LETROZOLE, LEUCOVORIN CALCIUM, LEUPROLIDE ACETATE, LEVOLEUCOVORIN, LEVOLEUCOVORIN CALCIUM, LOMUSTINE, LORLATINIB, LURBINECTEDIN, LUSPATERCEPT-AAMT, LUTETIUM LU 177 DOTATATE, MEDROXYPROGESTERONE ACETATE, MEGESTROL ACETATE, MELPHALAN, MERCAPTOPURINE, MESNA, METHOTREXATE, METHOTREXATE SODIUM, METHOXSALEN, METHYLPREDNISOLONE, METHYLPREDNISOLONE ACETATE, METHYLPREDNISOLONE SODIUM SUCCINATE, METHYLTESTOSTERONE, MIDOSTAURIN, MIFEPRISTONE, MITOMYCIN, MITOTANE, MOGAMULIZUMAB-KPKC, MOXETUMOMAB PASUDOTOX-TDFK, NABILONE, NECITUMUMAB, NELARABINE, NILUTAMIDE, NIVOLUMAB, OBINUTUZUMAB, OCTREOTIDE ACETATE, OFATUMUMAB, OLAPARIB, OLARATUMAB, OMACETAXINE MEPESUCCINATE, ONDANSETRON, ONDANSETRON HCL, OXALIPLATIN, OXYMETHOLONE, PACLITAXEL, PALBOCICLIB, PALIFERMIN, PAMIDRONATE DISODIUM, PANITUMUMAB,

	PEGASPARGASE, PEGFILGRASTIM, PEGFILGRASTIM-BMEZ, PEGFILGRASTIM-CBQV, PEGINTERFERON ALFA-2A, PEGINTERFERON ALFA-2B, PEMBROLIZUMAB, PEMETREXED DISODIUM, PEMIGATINIB, PENTOSTATIN, PERTUZUMAB, PLERIXAFOR, PLICAMYCIN, POMALIDOMIDE, PORFIMER SODIUM, PRALATREXATE, PRALSETINIB, PREDNISOLONE, PREDNISOLONE ACETATE, PREDNISOLONE SODIUM PHOSPHATE, PREDNISONE, RAMUCIRUMAB, RASBURICASE, REGORAFENIB, RIPRETINIB, RITUXIMAB, RITUXIMAB-ABBS, RITUXIMAB-PVVR, ROMIDEPSIN, SACITUZUMAB GOVITECAN-HZIY, SAMARIUM SM 153 LEXIDRONAM, SARGRAMOSTIM, SELINEXOR, SELPERCATINIB, SILTUXIMAB, STREPTOZOCIN, SUNITINIB MALATE, TAFASITAMAB-CXIX, TAGRAXOFUSP-ERZS, TALIMOGENE LAHERPAREPVEC, TAMOXIFEN CITRATE, TBO-FILGRASTIM, TEMOZOLOMIDE, TEMSIROLIMUS, TENIPOSIDE, TESTOLACTONE, TESTOSTERONE ENANTHATE, THALIDOMIDE, THIOGUANINE, THIOTEPA, THYROTROPIN ALFA, TISAGENLECLEUCEL, TOREMIFENE CITRATE, TRABECTEDIN, TRASTUZUMAB, TRASTUZUMAB-ANNS, TRASTUZUMAB-DKST, TRASTUZUMAB-DTTB, TRASTUZUMAB-PKRB, TRASTUZUMAB-QYYP, TRETINOIN, TRIAMCINOLONE, TRIAMCINOLONE ACETONIDE, TRIAMCINOLONE HEXACETONIDE, TRILOSTANE, TRIMETREXATE GLUCURONATE, TRIPTORELIN PAMOATE, TUCATINIB, URACIL MUSTARD, URIDINE TRIACETATE, VALRUBICIN, VANDETANIB, VEMURAFENIB, VENETOCLAX, VINBLASTINE SULFATE, VINCRISTINE SULFATE, VINORELBINE TARTRATE, VISMODEGIB, VORINOSTAT, ZANUBRUTINIB, ZIV-AFLIBERCEPT, ZOLEDRONIC ACID<br>OR<br>A pharmacy claim with at least one of the following cancer therapy brand names: A-HYDROCORT, A-METHAPRED, ABIRATERONE ACETATE, ABRAXANE, ABSORICA, ABSORICA LD, ACCUTANE, ACTIMMUNE, ADCETRIS, ADRIAMYCIN, ADRUCIL, AFINITOR, AFINITOR DISPERZ, AGRYLIN, AKYNZEO, ALDARA, ALECENSA, ALIMTA, ALIQOPA, ALKERAN, ALOXI, ALUNBRIG, AMIFOSTINE, AMNESTEEM, ANADROL-50, ANASTROZOLE, ANDROID, ANDROXY, ANZEMET, APREPITANT, ARANESP, ARIMIDEX, ARISTOSPAN, AROMASIN, ARRANON, ARSENIC TRIOXIDE, ARZERRA, ASPARLAS, AVASTIN, AVODART, AYVAKIT, AZACITIDINE, BALVERSA, BAVENCIO, BAYCADRON, BELEODAQ, BELRAPZO, BENDEKA, BESPONSA, BEXAROTENE, BEXXAR, BICALUTAMIDE, BICNU, BLENREP, BLINCYTO, BORTEZOMIB, BOSULIF, BRAFTOVI, BROMOCRIPTINE MESYLATE, BRUKINSA, BUSULFAN, BUSULFEX, CABOMETYX, CALQUENCE, CAMPATH, CAMPTOSAR, CAPECITABINE, CAPRELSA, CARBOPLATIN, CARMUSTINE, CASODEX, CEENU, CERVARIX, CESAMET, CINVANTI, CISPLATIN, CLADRIBINE, CLARAVIS, CLOFARABINE, CLOLAR, COMETRIQ, COPIKTRA, CORTEF, CORTISONE ACETATE, COSMEGEN, COTELLIC, CYCLOPHOSPHAMIDE, CYCLOSPORINE, CYCLOSPORINE MODIFIED, CYRAMZA, CYSTAGON, CYTARABINE, CYTOXAN, DACARBAZINE, DACOGEN, DACTINOMYCIN, DARZALEX, DARZALEX FASPRO, DAUNOXOME, DAURISMO, DECADRON, DECITABINE, DELATESTRYL, DELESTROGEN, DELTASONE, DEPO-MEDROL, DEPO-PROVERA, DEPOCYT, DEXABLISS, DEXAMETHASONE, DEXAMETHASONE INTENSOL, DEXAMETHASONE SODIUM PHOSPHATE, DEXASONE, DEXRAZOXANE, DIDRONEL, DOCEFREZ, DOCETAXEL, DOXIL, DRONABINOL, DUTASTERIDE, DXEVO, EFUDEX, ELIGARD, ELITEK, ELLENCE, ELOXATIN, ELSPAR, ELZONRIS, EMCYT, EMEND, EMPLICITI, ENHERTU, EPOGEN, ERBITUX, ERGAMISOL, ERIVEDGE, ERLEADA, ERWINAZE, ESTRACE, ESTRADIOL, ESTRADIOL VALERATE, ETHYOL, ETOPOPHOS, ETOPOSIDE, EVEROLIMUS, EVISTA, EVOMELA, EXEMESTANE, FARESTON, FARYDAK, FASLODEX, FEMARA, FINASTERIDE, FIRMAGON, FLO-PRED, FLOXURIDINE, FLUDARA, FLUDARABINE PHOSPHATE, FLUOROURACIL, FLUTAMIDE, FOLOTYN, FOSAPREPITANT DIMEGLUMINE, FULPHILA, FULVESTRANT, FUSILEV, GARDASIL, GARDASIL 9, GAVRETO, GAZYVA, GEMZAR, GENGRAF, GILOTRIF, GLEEVEC, GLEOSTINE, GLIADEL, GRANISOL, GRANIX, HALAVEN, HEMADY, HERCEPTIN, HERCEPTIN HYLECTA, HERZUMA, HEXALEN, HICON, HIZENTRA, HYCAMTIN, HYDREA, HYDROCORTISONE, HYDROCORTONE, HYDROXYPROGESTERONE CAPROATE, HYDROXYUREA, IBRANCE, ICLUSIG, IDAMYCIN, IDAMYCIN PFS, IDHIFA, IFEX, IFOSFAMIDE, IMATINIB MESYLATE, IMBRUVICA, IMFINZI, IMIQUIMOD, IMLYGIC, INFUGEM, INLYTA, INQOVI, INREBIC, INTRON A, IRESSA, ISOTRETINOIN, ISTODAX, IXEMPRA, JAKAFI, JELMYTO, JEVTANA, KADCYLA, KANJINTI, KENALOG-10, KENALOG-40, KENALOG-80, KEPIVANCE, KEYTRUDA, KHAPZORY, KISQALI, KISQALI FEMARA CO-PACK, KORLYM, KOSELUGO, KYMRIAH, KYPROLIS, KYTRIL, LAPATINIB, LARTRUVO, LEMTRADA, LENVIMA, LETROZOLE, LEUCOVORIN CALCIUM, LEUKERAN, LEUKINE, LEUPROLIDE ACETATE, LEVOLEUCOVORIN CALCIUM, LIBTAYO, LIPODOX 50, LOMUSTINE, LONSURF, LORBRENA, LUMOXITI, LUPRON DEPOT, LUTATHERA, LYNPARZA, LYSODREN, MAKENA, MARINOL, MARQIBO, MATULANE, MAVENCLAD, MEDROL, MEDROXYPROGESTERONE ACETATE, MEGACE, MEGESTROL ACETATE, MEKINIST, MEKTOVI, MELPHALAN, MENEST, MERCAPTOPURINE, MESNA, MESNEX, METASTRON, METHITEST, METHOTREXATE, METHOTREXATE SODIUM, METHYLPRED DP, METHYLPREDNISOLONE, METHYLPREDNISOLONE ACETATE, METHYLTESTOSTERONE, METOCLOPRAMIDE HYDROCHLORIDE, MILLIPRED, MITOMYCIN, MITOSOL, MONJUVI, MOZOBIL, MUSTARGEN, MUTAMYCIN, MVASI, MYLERAN, MYLOTARG, MYORISAN, NAVELBINE, NEORAL, NERLYNX, NEULASTA, NEUPOGEN, NEXAVAR, NILANDRON, NILUTAMIDE, NINLARO, NIPENT, NIVESTYM, NOLVADEX, NUBEQA, OCTREOTIDE ACETATE, ODOMZO, OFEV, OGIVRI, ONCASPAR, ONDANSETRON HCL, ONIVYDE, ONTAK, ONTRUZANT, ONUREG, OPDIVO, ORAPRED, ORAPRED ODT, OXALIPLATIN, PACLITAXEL, PADCEV, PAMIDRONATE DISODIUM, PANRETIN, PARAPLATIN, PARLODEL, PEDIAPRED, PEGASYS, PEMAZYRE, PERJETA, PHESGO, PHOTOFRIN, PIQRAY,

	PLATINOL-AQ, PLENAXIS, POLIVY, POMALYST, PORTRAZZA, POTELIGEO, PREDNISOLONE, PREDNISOLONE SODIUM PHOSPHATE, PREDNISONE, PREDNISONE INTENSOL, PREMARIN, PROCRIT, PROCYSBI, PROLEUKIN, PROLIA, PROSCAR, PROVENGE, PROVERA, PURINETHOL, PURIXAN, QINLOCK, QUADRAMET, RAYOS, READYSHARP DEXAMETHASONE, READYSHARP TRIAMCINOLONE, REBLOZYL, REGLAN, RETACRIT, RETEVMO, REVLIMID, RITUXAN, RITUXAN HYCELA, ROMIDEPSIN, ROZLYTREK, RUBEX, RUBRACA, RUXIENCE, RYDAPT, SANCUSO, SANDIMMUNE, SANDOSTATIN, SANDOSTATIN LAR, SANDOSTATIN LAR DEPOT, SARCLISA, SODIUM IODIDE I-131, SOLTAMOX, SOLU-CORTEF, SOLU-MEDROL, SOMATULINE DEPOT, SPRYCEL, STIVARGA, SUPPRELIN LA, SUSTOL, SUTENT, SYLATRON, SYLVANT, SYMPROIC, SYNDROS, SYNRIBO, TABLOID, TABRECTA, TAFINLAR, TAGRISSO, TALZENNA, TAMOXIFEN CITRATE, TARCEVA, TARGRETIN, TASIGNA, TAXOL, TAXOTERE, TAZVERIK, TECARTUS, TECENTRIQ, TEMODAR, TEMOZOLOMIDE, TEMSIROLIMUS, TENIPOSIDE, TEPADINA, TESLAC, TESTOSTERONE ENANTHATE, TESTRED, THALOMID, THERACYS, THIOTEPA, THYROGEN, TIBSOVO, TICE BCG, TOPOSAR, TOREMIFENE CITRATE, TORISEL, TOTECT, TRAZIMERA, TREANDA, TRELSTAR, TRETINOIN, TREXALL, TRIAMCINOLONE ACETONIDE, TRISENOX, TRODELVY, TRUXIMA, TUKYSA, TURALIO, TYKERB, UDENYCA, UNITUXIN, UVADEX, VALCHLOR, VALSTAR, VANDETANIB, VANTAS, VARUBI, VECTIBIX, VELCADE, VENCLEXTA, VERIPRED 20, VERZENIO, VESANOID, VIADUR, VIDAZA, VINBLASTINE SULFATE, VINCASAR PFS, VINCRISTINE SULFATE, VINORELBINE TARTRATE, VISTOGARD, VITRAKVI, VIZIMPRO, VORAXAZE, VOTRIENT, VYXEOS, XALKORI, XATMEP, XELODA, XGEVA, XOFIGO, XOSPATA, XPOVIO, XTANDI, XURIDEN, YERVOY, YESCARTA, YONDELIS, YONSA, ZALTRAP, ZANOSAR, ZARXIO, ZCORT, ZEJULA, ZELBORAF, ZENATANE, ZEPZELCA, ZEVALIN, ZIEXTENZO, ZINECARD, ZIRABEV, ZODEX, ZOFRAN, ZOFRAN ODT, ZOLADEX, ZOLEDRONIC ACID, ZOLINZA, ZOMETA, ZUPLENZ, ZYDELIG, ZYKADIA, ZYTIGA<br>OR<br>A medical of pharmacy claim with at least one of the following cancer therapy HCPCS or CPT codes: A9545, C9021, C9025, C9058, C9062, C9064, C9117, C9213, C9215, C9235, C9243, C9257, C9259, C9260, C9273, C9284, C9287, C9295, C9297, C9415, C9421, C9423, C9429, C9431, C9432, C9433, C9440, C9455, C9483, J0128, J0640, J0641, J0642, J0885, J0896, J0897, J1030, J1040, J1050, J1051, J1260, J1380, J1436, J1440, J1446, J1453, J1675, J1710, J1720, J1725, J1930, J1950, J2405, J2469, J2502, J2505, J2650, J2783, J3120, J3121, J3305, J7509, J7512, J7527, J8520, J8530, J8540, J8560, J8562, J8600, J8655, J8670, J8700, J8705, J8999, J9001, J9015, J9017, J9019, J9020, J9025, J9030, J9032, J9033, J9043, J9050, J9055, J9060, J9062, J9092, J9093, J9095, J9096, J9098, J9120, J9140, J9151, J9160, J9171, J9176, J9180, J9181, J9185, J9190, J9198, J9200, J9202, J9203, J9204, J9207, J9211, J9225, J9246, J9262, J9263, J9264, J9266, J9268, J9269, J9270, J9290, J9291, J9295, J9301, J9303, J9305, J9307, J9308, J9310, J9320, J9325, J9328, J9330, J9358, J9360, J9370, J9371, J9380, J9390, J9395, Q0162, Q0166, Q0179, Q2025, Q2043, Q2048, Q2050, Q5101, Q5111, Q5113, Q5115, Q5117, Q5119, Q5120, S0108, S0115, S0116, S0119, S0146, S0156, S0174, S0176, S0177, S0178, S0179, S0181, S2107, S9338, A9534, C1086, C9004, C9012, C9027, C9065, C9066, C9110, C9118, C9127, C9131, C9205, C9210, C9214, C9216, C9237, C9239, C9240, C9252, C9253, C9262, C9265, C9276, C9280, C9289, C9292, C9293, C9296, C9414, C9417, C9418, C9420, C9422, C9424, C9425, C9426, C9427, C9428, C9430, C9437, C9474, C9492, J0202, J0207, J0594, J0881, J0894, J1020, J1094, J1100, J1190, J1441, J1442, J1447, J1626, J1627, J1726, J1729, J2353, J2354, J2425, J2562, J2820, J2860, J2920, J2930, J3130, J3240, J3300, J3301, J3315, J3487, J3489, J3590, J7506, J7510, J7684, J8499, J8501, J8510, J8521, J8561, J8565, J8610, J8650, J9000, J9002, J9010, J9027, J9031, J9034, J9035, J9036, J9039, J9040, J9041, J9042, J9045, J9047, J9065, J9070, J9080, J9090, J9091, J9094, J9097, J9100, J9110, J9118, J9130, J9150, J9155, J9170, J9177, J9178, J9179, J9182, J9201, J9206, J9208, J9209, J9213, J9214, J9216, J9217, J9218, J9219, J9226, J9227, J9228, J9230, J9245, J9250, J9260, J9261, J9265, J9267, J9271, J9280, J9285, J9293, J9299, J9300, J9302, J9304, J9306, J9313, J9315, J9340, J9350, J9351, J9354, J9355, J9356, J9357, J9375, J9400, J9600, J9999, Q0167, Q0168, Q0180, Q2017, Q2024, Q2049, Q2051, Q5114, Q5118, S0087, S0088, S0091, S0145, S0165, S0170, S0172, S0182, S0187, S0190
Primary immunodeficiency	Any prior history of a primary immunodeficiency defined as a medical claim with any of the following ICD-10 diagnosis codes: B20, D82, D82.0, D82.1, D82.3, D82.4, D82.9, B97.35, D45, D46.22, D47.1, D47.4, D47.9, D47.Z1, D47.Z9, D61.82, D75.81, D82.2, D82.8, R75, Z21
HIV infection	Any prior history of HIV defined as having as any medical claim with one of the following ICD-10 diagnosis codes: B20, B97.35, R75, Z21<br>OR<br>Any medical claim with the following HCPCS code: 3490F
Immunosuppressive therapy	A fill or claim for one of the following immunosuppressive therapies in the 60 days prior to index, defined as any pharmacy claim with at least one of the following generic names:<br>BELATACEPT, BRODALUMAB, CERTOLIZUMAB PEGOL, CYCLOSPORINE/CHONDROITIN SULFATE A SODIUM, CYTARABINE LIPOSOME/PF, DAUNORUBICIN/CYTARABINE LIPOSOMAL, DECITABINE/CEDAZURIDINE, EFALIZUMAB, EMAPALUMAB-LZSG, INFLIXIMAB-DYYB, NATALIZUMAB, OCRELIZUMAB, PEMETREXED DISODIUM, RAVULIZUMAB-CWVZ, RILONACEPT, TACROLIMUS IN VEHICLE BASE NO.238, TACROLIMUS/NIACINAMIDE, TERIFLUNOMIDE, TILDRAKIZUMAB-ASMN, TOCILIZUMAB, UPADACITINIB, ANAKINRA, ECULIZUMAB, FINGOLIMOD HCL, FLUOROURACIL/ADHESIVE BANDAGE, INEBILIZUMAB-CDON, INFLIXIMAB, INFLIXIMAB-AXXQ,

	NELARABINE, OZANIMOD HYDROCHLORIDE, SARILUMAB, SATRALIZUMAB-MWGE, SECUKINUMAB, SIPONIMOD, STREPTOZOCIN, TACROLIMUS, MICRONIZED, TACROLIMUS/HYALURONATE SODIUM/NIACINAMIDE, TEPROTUMUMAB-TRBW, VEDOLIZUMAB, ABATACEPT, ABATACEPT/MALTOSE, ALEFACEPT, AZATHIOPRINE SODIUM, BARICITINIB, BASILIXIMAB, CARMUSTINE IN POLIFEPROSAN 20, DIROXIMEL FUMARATE, GUSELKUMAB, INFLIXIMAB-ABDA, PRALATREXATE, SILTUXIMAB, TACROLIMUS ANHYDROUS, USTEKINUMAB, CANAKINUMAB/PF, GOLIMUMAB, MELPHALAN HCL/BETADEX SULFOBUTYL ETHER SODIUM, MUROMONAB-CD3, PIRFENIDONE, RISANKIZUMAB-RZAA, TOFACITINIB CITRATE, ALEMTUZUMAB, CLOFARABINE, GEMCITABINE HCL IN 0.9 % SODIUM CHLORIDE, POMALIDOMIDE, APREMILAST, BENDAMUSTINE HCL, DACLIZUMAB, IXEKIZUMAB, FLOXURIDINE, OFATUMUMAB, IFOSFAMIDE/MESNA, LOMUSTINE, DIMETHYL FUMARATE, MYCOPHENOLATE MOFETIL HCL, BELIMUMAB, CARMUSTINE, MELPHALAN, TEMSIROLIMUS, THIOTEPA, LENALIDOMIDE, THALIDOMIDE, CLADRIBINE, FLUDARABINE PHOSPHATE, ADALIMUMAB, CHLORAMBUCIL, MELPHALAN HCL, DECITABINE, METHOTREXATE, CYTARABINE/PF, ETANERCEPT, AZACITIDINE, IFOSFAMIDE, BUSULFAN, LEFLUNOMIDE, DACARBAZINE, MERCAPTOPURINE, MYCOPHENOLATE SODIUM, METHOTREXATE/PF, CYTARABINE, EVEROLIMUS, SIROLIMUS, CYCLOSPORINE, MODIFIED, CAPECITABINE, CYCLOSPORINE<br>OR<br>Any medical claim with at least one of the following CPT/HCPC procedure codes: 80158, 80180, 80197, C9106, C9126, C9211, C9212, C9230, C9236, C9239, C9249, C9261, C9264, C9436, C9438, C9455, J0129, J0215, J1300, J1438, J1602, J1628, J2350, J3357, J7501, J7502, J7504, J7507, J7508, J7513, J7517, J7518, J7525, J7527, J8561, J8610, J9070, J9080, J9090, J9092, J9093, J9096, J9250, J9260, J9311, J9312, K0119, K0122, K0412, Q5103, S0087, S0162, S9359, 80169, 80195, C9006, C9020, C9026, C9110, C9219, C9286, C9419, C9420, C9421, J0135, J0202, J0480, J0485, J0490, J0638, J0717, J0718, J1745, J2323, J2793, J2860, J3245, J3262, J3358, J3380, J7500, J7503, J7505, J7511, J7515, J7520, J8530, J9010, J9065, J9091, J9094, J9095, J9097, J9310, J9330, K0120, K0121, K0123, Q2019, Q2044, Q4079, Q5104, Q5109, S0193
Other immunocompromising conditions	The occurrence of a medical claim with the following attributes during the baseline period: Diagnosis Code, ICD-10 is any of: D84.9, D81.1, D84.81
**Vulnerable conditions**	
Age ≥65 years	Recorded age on index date ≥ 65 years
Stage 4/5 chronic kidney disease or end-stage renal disease	The occurrence of a medical claim during with the following attributes during the baseline period:<br>Diagnosis Code, ICD-10 is any of: I13.11, N18.6,I12.0, I13.2,N18.4, N18.5
Chronic liver disease	The occurrence of a medical claim with the following attributes during the baseline period:<br>Diagnosis Code, ICD-10 is any of: B18, B18.0, B18.1, K70.11, K71.50, K71.7, K73.0, K74.6, K74.60, K76.2, K70.10, K74.1, K74.2, K74.5, K74.60, K76.2, K74.1, K74.2, K74.5, K70.30, K71.4, K71.51, K73, K73.8, K73.9, K74, K74.3, K76.81, B18.9, K70.2, K70.3, K73.1, K74.0, K74.69, B18.2, B18.8, K70.31, K71.5, K73.2, K74.4, K72.1, K72.10, K71.10, K72.11, K72.91, K76.7, K71.1, K71.11, K70.40, K72.9, K72.90, K70.4, K70.41
Chronic lung disease (moderate to severe asthma, bronchiectasis, bronchopulmonary dysplasia, COPD, as interstitial lung disease, pulmonary embolism, pulmonary hypertension)	The occurrence of a medical claim with the following attributes during the baseline period:<br>Diagnosis Code, ICD-10 is any of: J45.51, J45.52, J45.40, J45.41, J45.42, J45.50, J41.0, J41.8, J43.0, J43.1, J43.2, J43.8, J44.0, J44.1, J44.9, J41.1, J42, J43.9, J84, J84.1, J84.17,, J84.8, J84.89, J84.9
Diabetes (type 1 or type 2)	The occurrence of a medical claim with the following attributes during the baseline period:<br>Diagnosis Code, ICD-10 is any of:E10.x and E11.x
Heart conditions (heart failure, coronary artery disease, cardiomyopathies, and (hypertension))	The occurrence of a medical claim with the following attributes during the baseline period:<br>Diagnosis Code, ICD-10 is any of: I10.x-I15.x, I20.x-I25.x, I50.x
Obesity	The occurrence of a medical claim with the following attributes during the baseline period:<br>Diagnosis Code, ICD-10 is any of: E66.0, E66.01, E66.09, E66.2, E66.8, E66.9
Pregnancy	The occurrence of a medical claim with the following attributes during the baseline period:<br>Diagnosis Code, ICD-10 is any of: O00.01, O00.109, O00.11, O00.2, O00.20, O00.202, O00.209, O00.21,

	O00.211, O00.80, O00.90, O00.91, O01.0, O01.9, O02.1, O02.89, O02.9, O03.1, O03.2, O03.32, O03.33, O03.34, O03.35, O03.37, O03.38, O03.4, O03.5, O03.7, O03.81, O03.82, O03.83, O03.85, O03.87, O04.5, O04.82, O04.83, O04.84, O04.85, O04.87, O07.0, O07.1, O07.2, O07.33, O07.34, O08.2, O08.3, O08.4, O08.5, O08.81, O08.82, O08.89, O08.9, O09.01, O09.02, O09.13, O09.213, O09.292, O09.299, O09.31, O09.32, O09.33, O09.42, O09.521, O09.523, O09.619, O09.622, O09.623, O09.71, O09.72, O09.73, O09.811, O09.813, O09.819, O09.821, O09.829, O09.891, O09.893, O09.899, O09.A0, O09.A3, O10.013, O10.019, O10.02, O10.03, O10.111, O10.113, O10.119, O10.22, O10.312, O10.313, O10.33, O10.412, O10.413, O10.42, O10.911, O10.913, O10.919, O10.92, O11.2, O11.3, O11.4, O11.9, O12.00, O12.01, O12.04, O12.11, O12.12, O12.13, O12.14, O12.20, O12.21, O12.23, O13.2, O13.3, O13.5, O14.02, O14.05, O14.10, O14.13, O14.14, O14.15, O14.22, O14.24, O14.25, O14.92, O14.93, O15.03, O15.9, O16.1, O16.9, O21.0, O21.2, O22.02, O22.11, O22.12, O22.13, O22.30, O22.31, O22.33, O22.40, O22.42, O22.43, O22.51, O22.53, O22.8X1, O22.8X2, O22.90, O22.91, O22.93, O23.01, O23.02, O23.03, O23.10, O23.11, O23.12, O23.21, O23.30, O23.32, O23.33, O23.40, O23.41, O23.42, O23.592, O23.593, O23.92, O24.011, O24.012, O24.019, O24.02, O24.03, O24.113, O24.119, O24.12, O24.13, O24.311, O24.313, O24.319, O24.414, O24.434, O24.435, O24.811, O24.812, O24.813, O24.819, O24.82, O24.912, O24.913, O24.92, O25.11, O25.13, O25.3, O26.11, O26.13, O26.20, O26.23, O26.30, O26.31, O26.32, O26.50, O26.52, O26.53, O26.611, O26.612, O26.619, O26.62, O26.63, O26.711, O26.712, O26.719, O26.73, O26.811, O26.819, O26.821, O26.832, O26.833, O26.841, O26.849, O26.852, O26.853, O26.86, O26.872, O26.892, O26.893, O26.91, O26.92, O28.3, O28.4, O28.5, O28.9, O29.012, O29.092, O29.099, O29.111, O29.112, O29.119, O29.122, O29.123, O29.191, O29.199, O29.292, O29.299, O29.3X1, O29.3X2, O29.3X3, O29.3X9, O29.40, O29.5X2, O29.5X3, O29.5X9, O29.60, O29.62, O29.63, O29.8X2, O29.8X3, O29.90, O29.91, O30.001, O30.002, O30.011, O30.029, O30.039, O30.042, O30.091, O30.093, O30.103, O30.112, O30.121, O30.122, O30.123, O30.129, O30.131, O30.139, O30.192, O30.193, O30.199, O30.201, O30.202, O30.203, O30.211, O30.212, O30.213, O30.219, O30.223, O30.229, O30.292, O30.293, O30.801, O30.802, O30.803, O30.812, O30.813, O30.821, O30.822, O30.823, O30.829, O30.831, O30.832, O30.833, O30.839, O30.891, O30.90, O30.91, O31.00X1, O31.00X2, O31.00X3, O31.00X5, O31.00X9, O31.01X1, O31.01X2, O31.01X3, O31.01X4, O31.01X5, O31.01X9, O31.02X1, O31.02X3, O31.02X9, O31.03X0, O31.03X2, O31.03X3, O31.03X4, O31.10X0, O31.10X1, O31.10X3, O31.10X9, O31.11X0, O31.11X2, O31.11X3, O31.11X4, O31.11X9, O31.12X3, O31.12X5, O31.12X9, O31.13X0, O31.13X2, O31.13X5, O31.20X0, O31.20X1, O31.20X2, O31.20X3, O31.20X5, O31.21X0, O31.21X4, O31.21X9, O31.22X1, O31.22X2, O31.22X3, O31.22X5, O31.22X9, O31.23X1, O31.23X2, O31.23X3, O31.23X4, O31.23X5, O31.23X9, O31.30X0, O31.30X1, O31.30X5, O31.30X9, O31.31X0, O31.31X1, O31.31X2, O31.31X4, O31.31X9, O31.32X1, O31.32X3, O31.32X4, O31.32X9, O31.33X1, O31.33X2, O31.33X3, O31.33X4, O31.33X9, O31.8X13, O31.8X14, O31.8X15, O31.8X19, O31.8X21, O31.8X24, O31.8X25, O31.8X29, O31.8X33, O31.8X90, O31.8X91, O31.8X95, O31.8X99, O32.0XX1, O32.0XX2, O32.0XX3, O32.0XX4, O32.1XX1, O32.1XX5, O32.1XX9, O32.2XX0, O32.2XX3, O32.2XX5, O32.2XX9, O32.3XX0, O32.3XX1, O32.3XX2, O32.3XX3, O32.4XX1, O32.4XX4, O32.4XX9, O32.6XX9, O32.8XX0, O32.8XX4, O32.8XX5, O32.8XX9, O32.9XX2, O32.9XX9, O33.0, O33.3XX0, O33.3XX1, O33.3XX2, O33.3XX4, O33.4XX1, O33.4XX4, O33.4XX9, O33.5XX0, O33.5XX2, O33.5XX3, O33.5XX4, O33.6XX0, O33.6XX1, O33.6XX3, O33.6XX4, O33.6XX5, O33.6XX9, O33.7, O33.7XX0, O33.7XX3, O33.7XX4, O33.7XX5, O33.8, O34.01, O34.02, O34.10, O34.12, O34.13, O34.212, O34.22, O34.31, O34.33, O34.512, O34.521, O34.523, O34.531, O34.533, O34.591, O34.592, O34.593, O34.599, O34.61, O34.63, O34.70, O34.71, O34.73, O34.83, O34.90, O35.0XX1, O35.0XX2, O35.1XX5, O35.1XX9, O35.2XX0, O35.2XX2, O35.2XX3, O35.2XX5, O35.2XX9, O35.3XX0, O35.3XX2, O35.3XX3, O35.3XX9, O35.4XX1, O35.4XX3, O35.4XX4, O35.4XX5, O35.4XX9, O35.5XX0, O35.5XX3, O35.5XX4, O35.5XX5, O35.6XX0, O35.6XX2, O35.6XX5, O35.7XX1, O35.7XX2, O35.7XX3, O35.7XX4, O35.7XX5, O35.8XX1, O35.8XX2, O35.8XX4, O35.9XX3, O35.9XX4, O36.0110, O36.0114, O36.0115, O36.0119, O36.0120, O36.0122, O36.0123, O36.0125, O36.0129, O36.0133, O36.0134, O36.0135, O36.0139, O36.0191, O36.0195, O36.0911, O36.0912, O36.0913, O36.0914, O36.0915, O36.0921, O36.0922, O36.0923, O36.0929, O36.0931, O36.0935, O36.0939, O36.0992, O36.0993, O36.0994, O36.0995, O36.1110, O36.1113, O36.1114, O36.1120, O36.1129, O36.1130, O36.1132, O36.1133, O36.1139, O36.1191, O36.1193, O36.1910, O36.1911, O36.1914, O36.1919, O36.1920, O36.1923, O36.1932, O36.1934, O36.1935, O36.1990, O36.1992, O36.20X3, O36.20X4, O36.20X9, O36.21X0, O36.21X1, O36.21X3, O36.21X4, O36.21X5, O36.22X1, O36.22X3, O36.22X4, O36.22X5, O36.22X9, O36.23X0, O36.23X2, O36.23X3, O36.23X5, O36.4XX0, O36.4XX1, O36.4XX2, O36.4XX4, O36.4XX9, O36.5112, O36.5113, O36.5114, O36.5115, O36.5120, O36.5121, O36.5123, O36.5124, O36.5130, O36.5132, O36.5133, O36.5134, O36.5139, O36.5193, O36.5194, O36.5199, O36.5911, O36.5913, O36.5914, O36.5920, O36.5922, O36.5923, O36.5924, O36.5931, O36.5935, O36.5939, O36.5992, O36.5993, O36.5994, O36.5995, O36.5999, O36.60X0, O36.60X2, O36.60X3, O36.60X5, O36.61X0, O36.61X4, O36.61X5, O36.61X9, O36.62X4, O36.62X5, O36.63X1, O36.63X2, O36.63X3, O36.63X5, O36.63X9, O36.70X0, O36.70X1, O36.70X4, O36.71X0, O36.71X1, O36.71X2, O36.71X3, O36.71X9, O36.72X0, O36.72X5, O36.73X1, O36.73X4, O36.80X2, O36.80X3, O36.80X9, O36.8121, O36.8123, O36.8124, O36.8131, O36.8132, O36.8135, O36.8190, O36.8193, O36.8195, O36.8210, O36.8213, O36.8219, O36.8221, O36.8222, O36.8223, O36.8224, O36.8225, O36.8229, O36.8230, O36.8290, O36.8291, O36.8294, O36.8299, O36.8310, O36.8311, O36.8313, O36.8314, O36.8320, O36.8321, O36.8322, O36.8323, O36.8324, O36.8329, O36.8330, O36.8331, O36.8332, O36.8335, O36.8391, O36.8392, O36.8395, O36.8399, O36.8910, O36.8911, O36.8919, O36.8920, O36.8922, O36.8923, O36.8924, O36.8925, O36.8931, O36.8932, O36.8934, O36.8935, O36.8939, O36.8992, O36.8993, O36.8999, O36.90X0,

	O36.90X1, O36.90X2, O36.90X4, O36.90X9, O36.91X2, O36.91X5, O36.92X1, O36.92X2, O36.93X2, O40.1XX0, O40.1XX2, O40.2XX1, O40.2XX2, O40.2XX3, O40.3XX2, O40.3XX3, O40.3XX4, O40.3XX9, O40.9XX0, O40.9XX2, O40.9XX4, O40.9XX5, O40.9XX9, O41.00X1, O41.00X3, O41.00X5, O41.00X9, O41.01X0, O41.01X1, O41.01X9, O41.02X1, O41.02X2, O41.03X5, O41.1010, O41.1011, O41.1012, O41.1014, O41.1015, O41.1021, O41.1022, O41.1023, O41.1024, O41.1030, O41.1031, O41.1032, O41.1034, O41.1039, O41.1091, O41.1093, O41.1094, O41.1099, O41.1210, O41.1211, O41.1219, O41.1222, O41.1229, O41.1232, O41.1234, O41.1290, O41.1293, O41.1294, O41.1295, O41.1410, O41.1413, O41.1414, O41.1420, O41.1421, O41.1422, O41.1423, O41.1424, O41.1429, O41.1431, O41.1433, O41.1499, O41.8X10, O41.8X11, O41.8X12, O41.8X15, O41.8X21, O41.8X23, O41.8X24, O41.8X25, O41.8X30, O41.8X33, O41.8X34, O41.8X35, O41.8X39, O41.8X90, O41.8X92, O41.8X93, O41.8X99, O41.90X0, O41.90X1, O41.90X2, O41.90X4, O41.90X9, O41.91X1, O41.91X9, O41.92X2, O41.92X5, O41.93X0, O41.93X1, O41.93X3, O41.93X4, O41.93X5, O41.93X9, O42.013, O42.02, O42.10, O42.111, O42.119, O42.90, O42.911, O42.913, O43.022, O43.103, O43.112, O43.113, O43.123, O43.191, O43.192, O43.193, O43.211, O43.219, O43.221, O43.232, O43.811, O43.812, O43.813, O43.891, O43.91, O43.92, O43.93, O44.10, O44.12, O44.13, O44.22, O44.30, O44.31, O44.33, O44.42, O44.43, O44.50, O44.51, O44.53, O45.001, O45.002, O45.009, O45.013, O45.019, O45.022, O45.023, O45.091, O45.092, O45.099, O45.8X9, O45.92, O46.002, O46.003, O46.009, O46.012, O46.013, O46.019, O46.022, O46.023, O46.092, O46.099, O46.8X2, O46.90, O46.91, O46.92, O46.93, O47.02, O60.03, O60.10X1, O60.10X3, O60.10X4, O60.10X9, O60.12X0, O60.12X2, O60.12X3, O60.13X2, O60.13X3, O60.13X4, O60.13X5, O60.13X9, O60.14X1, O60.14X3, O60.14X4, O60.14X5, O60.14X9, O60.20X0, O60.20X4, O60.22X0, O60.22X2, O60.22X3, O60.22X5, O60.23X0, O60.23X1, O60.23X2, O60.23X3, O60.23X9, O61.1, O61.8, O62.8, O63.1, O63.2, O63.9, O64.0XX0, O64.1XX3, O64.1XX5, O64.2XX5, O64.2XX9, O64.3XX4, O64.4XX2, O64.4XX4, O64.4XX9, O64.5XX0, O64.5XX1, O64.5XX2, O64.5XX5, O64.8XX0, O64.8XX1, O64.9XX2, O64.9XX3, O64.9XX5, O64.9XX9, O65.0, O65.1, O65.3, O65.4, O65.5, O66.1, O66.2, O66.41, O67.0, O67.8, O68, O69.0XX0, O69.0XX1, O69.0XX4, O69.0XX9, O69.1XX0, O69.1XX1, O69.2XX1, O69.2XX4, O69.3XX0, O69.3XX2, O69.3XX4, O69.4XX2, O69.4XX4, O69.4XX5, O69.4XX9, O69.5XX0, O69.5XX3, O69.5XX4, O69.5XX5, O69.81X2, O69.81X3, O69.81X4, O69.82X0, O69.82X1, O69.82X4, O69.82X9, O69.89X0, O69.89X1, O69.9XX1, O69.9XX3, O69.9XX5, O69.9XX9, O70.20, O70.21, O70.22, O70.3, O70.4, O71.02, O71.3, O71.6, O71.7, O71.81, O71.82, O71.89, O72.0, O73.0, O73.1, O74.0, O74.4, O74.5, O75.0, O75.1, O75.3, O75.5, O75.89, O75.9, O76, O77.1, O77.8, O86.00, O86.02, O86.11, O86.13, O86.19, O86.20, O86.21, O86.22, O86.4, O86.81, O87.0, O87.1, O87.8, O87.9, O88.011, O88.012, O88.013, O88.02, O88.03, O88.112, O88.119, O88.12, O88.211, O88.213, O88.312, O88.313, O88.813, O89.09, O89.3, O89.4, O89.6, O89.8, O89.9, O90.1, O90.3, O90.5, O90.6, O90.81, O91.013, O91.111, O91.113, O91.12, O91.213, O91.219, O92.011, O92.012, O92.013, O92.02, O92.113, O92.119, O92.12, O92.13, O92.6, O92.79, O98.011, O98.012, O98.013, O98.02, O98.03, O98.113, O98.119, O98.13, O98.213, O98.22, O98.23, O98.319, O98.33, O98.413, O98.42, O98.43, O98.512, O98.519, O98.52, O98.611, O98.613, O98.619, O98.62, O98.63, O98.711, O98.712, O98.713, O98.719, O98.72, O98.911, O98.912, O98.913, O98.919, O98.92, O98.93, O99.011, O99.012, O99.013, O99.111, O99.112, O99.113, O99.211, O99.212, O99.215, O99.281, O99.284, O99.285, O99.313, O99.320, O99.323, O99.325, O99.330, O99.333, O99.340, O99.341, O99.342, O99.344, O99.353, O99.354, O99.355, O99.411, O99.413, O99.511, O99.513, O99.519, O99.52, O99.611, O99.612, O99.619, O99.712, O99.713, O99.810, O99.814, O99.815, O99.824, O99.830, O99.834, O99.835, O99.842, O99.844, O99.89, O99.893, O9A.111, O9A.112, O9A.113, O9A.12, O9A.13, O9A.211, O9A.213, O9A.23, O9A.311, O9A.313, O9A.319, O9A.411, O9A.413, O9A.419, O9A.42, O9A.43, O9A.513, O9A.519, Z32.01, Z33.1, Z34.03, Z34.81, Z34.82, Z34.90, Z34.91, Z34.92, Z34.93, Z36, Z36.1, Z36.3, Z36.4, Z36.5, Z36.84, Z36.87, Z36.88, Z36.89, Z36.8A, Z37.1, Z37.2, Z37.54, Z37.61, Z37.62, Z37.63, Z37.69, Z37.7, Z39.1, Z3A.08, Z3A.09, Z3A.18, Z3A.20, Z3A.23, Z3A.25, Z3A.26, Z3A.27, Z3A.28, Z3A.29, Z3A.31, Z3A.32, Z3A.33, Z3A.34, Z3A.37, Z3A.40, Z3A.41, Z3A.49, A34, O00.0, O00.00, O00.1, O00.10, O00.101, O00.102, O00.111, O00.112, O00.119, O00.201, O00.212, O00.219, O00.8, O00.81, O00.9, O01.1, O02.0, O02.81, O03.0, O03.30, O03.31, O03.36, O03.39, O03.6, O03.80, O03.84, O03.86, O03.88, O03.89, O03.9, O04.6, O04.7, O04.80, O04.81, O04.86, O04.88, O04.89, O07.30, O07.31, O07.32, O07.35, O07.36, O07.37, O07.38, O07.39, O07.4, O08.0, O08.1, O08.6, O08.7, O08.83, O09.00, O09.03, O09.10, O09.11, O09.12, O09.211, O09.212, O09.219, O09.291, O09.293, O09.30, O09.40, O09.41, O09.43, O09.511, O09.512, O09.513, O09.519, O09.522, O09.529, O09.611, O09.612, O09.613, O09.621, O09.629, O09.70, O09.812, O09.822, O09.823, O09.892, O09.90, O09.91, O09.92, O09.93, O09.A1, O09.A2, O10.011, O10.012, O10.112, O10.12, O10.13, O10.211, O10.212, O10.213, O10.219, O10.23, O10.311, O10.319, O10.32, O10.411, O10.419, O10.43, O10.912, O10.93, O11.1, O11.5, O12.02, O12.03, O12.05, O12.10, O12.15, O12.22, O12.24, O12.25, O13.1, O13.4, O13.9, O14.00, O14.03, O14.04, O14.12, O14.20, O14.23, O14.90, O14.94, O14.95, O15.00, O15.02, O15.1, O15.2, O16.2, O16.3, O16.4, O16.5, O20.0, O20.8, O20.9, O21.1, O21.8, O21.9, O22.00, O22.01, O22.03, O22.10, O22.20, O22.21, O22.22, O22.23, O22.32, O22.41, O22.50, O22.52, O22.8X3, O22.8X9, O22.92, O23.00, O23.13, O23.20, O23.22, O23.23, O23.31, O23.43, O23.511, O23.512, O23.513, O23.519, O23.521, O23.522, O23.523, O23.529, O23.591, O23.599, O23.90, O23.91, O23.93, O24.013, O24.111, O24.112, O24.312, O24.32, O24.33, O24.410, O24.415, O24.419, O24.420, O24.424, O24.425, O24.429, O24.430, O24.439, O24.83, O24.911, O24.919, O24.93, O25.10, O25.12, O25.2, O26.00, O26.01, O26.02, O26.03, O26.10, O26.12, O26.21, O26.22, O26.33, O26.40, O26.41, O26.42, O26.43, O26.51, O26.613, O26.713, O26.72, O26.812, O26.813, O26.822, O26.823, O26.829, O26.831, O26.839, O26.842, O26.843, O26.851, O26.859, O26.873, O26.879, O26.891, O26.899, O26.90, O26.93, O28.0, O28.1, O28.2, O28.8, O29.011, O29.013, O29.019, O29.021,

	O29.022, O29.023, O29.029, O29.091, O29.093, O29.113, O29.121, O29.129, O29.192, O29.193, O29.211, O29.212, O29.213, O29.219, O29.291, O29.293, O29.41, O29.42, O29.43, O29.5X1, O29.61, O29.8X1, O29.8X9, O29.92, O29.93, O30.003, O30.009, O30.012, O30.013, O30.019, O30.021, O30.022, O30.023, O30.031, O30.032, O30.033, O30.041, O30.043, O30.049, O30.092, O30.099, O30.101, O30.102, O30.109, O30.111, O30.113, O30.119, O30.132, O30.133, O30.191, O30.209, O30.221, O30.222, O30.231, O30.232, O30.233, O30.239, O30.291, O30.299, O30.809, O30.811, O30.819, O30.892, O30.893, O30.899, O30.92, O30.93, O31.00X0, O31.00X4, O31.01X0, O31.02X0, O31.02X2, O31.02X4, O31.02X5, O31.03X1, O31.03X5, O31.03X9, O31.10X2, O31.10X4, O31.10X5, O31.11X1, O31.11X5, O31.12X0, O31.12X1, O31.12X2, O31.12X4, O31.13X1, O31.13X3, O31.13X4, O31.13X9, O31.20X4, O31.20X9, O31.21X1, O31.21X2, O31.21X3, O31.21X5, O31.22X0, O31.22X4, O31.23X0, O31.30X2, O31.30X3, O31.30X4, O31.31X3, O31.31X5, O31.32X0, O31.32X2, O31.32X5, O31.33X0, O31.33X5, O31.8X10, O31.8X11, O31.8X12, O31.8X20, O31.8X22, O31.8X23, O31.8X30, O31.8X31, O31.8X32, O31.8X34, O31.8X35, O31.8X39, O31.8X92, O31.8X93, O31.8X94, O32.0XX0, O32.0XX5, O32.0XX9, O32.1XX0, O32.1XX2, O32.1XX3, O32.1XX4, O32.2XX1, O32.2XX2, O32.2XX4, O32.3XX4, O32.3XX5, O32.3XX9, O32.4XX0, O32.4XX2, O32.4XX3, O32.4XX5, O32.6XX0, O32.6XX1, O32.6XX2, O32.6XX3, O32.6XX4, O32.6XX5, O32.8XX1, O32.8XX2, O32.8XX3, O32.9XX0, O32.9XX1, O32.9XX3, O32.9XX4, O32.9XX5, O33.1, O33.2, O33.3XX3, O33.3XX5, O33.3XX9, O33.4XX0, O33.4XX2, O33.4XX3, O33.4XX5, O33.5XX1, O33.5XX5, O33.5XX9, O33.6XX2, O33.7XX1, O33.7XX2, O33.7XX9, O33.9, O34.00, O34.03, O34.11, O34.21, O34.211, O34.218, O34.219, O34.29, O34.30, O34.32, O34.40, O34.41, O34.42, O34.43, O34.511, O34.513, O34.519, O34.522, O34.529, O34.532, O34.539, O34.60, O34.62, O34.72, O34.80, O34.81, O34.82, O34.91, O34.92, O34.93, O35.0XX0, O35.0XX3, O35.0XX4, O35.0XX5, O35.0XX9, O35.1XX0, O35.1XX1, O35.1XX2, O35.1XX3, O35.1XX4, O35.2XX1, O35.2XX4, O35.3XX1, O35.3XX4, O35.3XX5, O35.4XX0, O35.4XX2, O35.5XX1, O35.5XX2, O35.5XX9, O35.6XX1, O35.6XX3, O35.6XX4, O35.6XX9, O35.7XX0, O35.7XX9, O35.8XX0, O35.8XX3, O35.8XX5, O35.8XX9, O35.9XX0, O35.9XX1, O35.9XX2, O35.9XX5, O35.9XX9, O36.0111, O36.0112, O36.0113, O36.0121, O36.0124, O36.0130, O36.0131, O36.0132, O36.0190, O36.0192, O36.0193, O36.0194, O36.0199, O36.0910, O36.0919, O36.0920, O36.0924, O36.0925, O36.0930, O36.0932, O36.0933, O36.0934, O36.0990, O36.0991, O36.0999, O36.1111, O36.1112, O36.1115, O36.1119, O36.1121, O36.1122, O36.1123, O36.1124, O36.1125, O36.1131, O36.1134, O36.1135, O36.1190, O36.1192, O36.1194, O36.1195, O36.1199, O36.1912, O36.1913, O36.1915, O36.1921, O36.1922, O36.1924, O36.1925, O36.1929, O36.1930, O36.1931, O36.1933, O36.1939, O36.1991, O36.1993, O36.1994, O36.1995, O36.1999, O36.20X0, O36.20X1, O36.20X2, O36.20X5, O36.21X2, O36.21X9, O36.22X0, O36.22X2, O36.23X1, O36.23X4, O36.23X9, O36.4XX3, O36.4XX5, O36.5110, O36.5111, O36.5119, O36.5122, O36.5125, O36.5129, O36.5131, O36.5135, O36.5190, O36.5191, O36.5192, O36.5195, O36.5910, O36.5912, O36.5915, O36.5919, O36.5921, O36.5925, O36.5929, O36.5930, O36.5932, O36.5933, O36.5934, O36.5990, O36.5991, O36.60X1, O36.60X4, O36.60X9, O36.61X1, O36.61X2, O36.61X3, O36.62X0, O36.62X1, O36.62X2, O36.62X3, O36.62X9, O36.63X0, O36.63X4, O36.70X2, O36.70X3, O36.70X5, O36.70X9, O36.71X4, O36.71X5, O36.72X1, O36.72X2, O36.72X3, O36.72X4, O36.72X9, O36.73X0, O36.73X2, O36.73X3, O36.73X5, O36.73X9, O36.80X0, O36.80X1, O36.80X4, O36.80X5, O36.8120, O36.8122, O36.8125, O36.8129, O36.8130, O36.8133, O36.8134, O36.8139, O36.8191, O36.8192, O36.8194, O36.8199, O36.8211, O36.8212, O36.8214, O36.8215, O36.8220, O36.8231, O36.8232, O36.8233, O36.8234, O36.8235, O36.8239, O36.8292, O36.8293, O36.8295, O36.8312, O36.8315, O36.8319, O36.8325, O36.8333, O36.8334, O36.8339, O36.8390, O36.8393, O36.8394, O36.8912, O36.8913, O36.8914, O36.8915, O36.8921, O36.8929, O36.8930, O36.8933, O36.8990, O36.8991, O36.8994, O36.8995, O36.90X3, O36.90X5, O36.91X0, O36.91X1, O36.91X3, O36.91X4, O36.91X9, O36.92X0, O36.92X3, O36.92X4, O36.92X5, O36.92X9, O36.93X0, O36.93X1, O36.93X3, O36.93X4, O36.93X5, O36.93X9, O40.1XX1, O40.1XX3, O40.1XX4, O40.1XX5, O40.1XX9, O40.2XX0, O40.2XX4, O40.2XX5, O40.2XX9, O40.3XX0, O40.3XX1, O40.3XX5, O40.9XX1, O40.9XX3, O41.00X0, O41.00X2, O41.00X4, O41.01X2, O41.01X3, O41.01X4, O41.01X5, O41.02X0, O41.02X3, O41.02X4, O41.02X5, O41.02X9, O41.03X0, O41.03X1, O41.03X2, O41.03X3, O41.03X4, O41.03X9, O41.1013, O41.1019, O41.1020, O41.1025, O41.1029, O41.1033, O41.1035, O41.1090, O41.1092, O41.1095, O41.1212, O41.1213, O41.1214, O41.1215, O41.1220, O41.1221, O41.1223, O41.1224, O41.1225, O41.1230, O41.1231, O41.1233, O41.1235, O41.1239, O41.1291, O41.1292, O41.1299, O41.1411, O41.1412, O41.1415, O41.1419, O41.1425, O41.1430, O41.1432, O41.1434, O41.1435, O41.1439, O41.1490, O41.1491, O41.1492, O41.1493, O41.1494, O41.1495, O41.8X13, O41.8X14, O41.8X19, O41.8X20, O41.8X22, O41.8X29, O41.8X31, O41.8X32, O41.8X91, O41.8X94, O41.8X95, O41.90X3, O41.90X5, O41.91X0, O41.91X2, O41.91X3, O41.91X4, O41.91X5, O41.92X0, O41.92X1, O41.92X3, O41.92X4, O41.92X9, O41.93X2, O42.00, O42.011, O42.012, O42.019, O42.112, O42.113, O42.12, O42.912, O42.919, O42.92, O43.011, O43.012, O43.013, O43.019, O43.021, O43.023, O43.029, O43.101, O43.102, O43.109, O43.111, O43.119, O43.121, O43.122, O43.129, O43.199, O43.212, O43.213, O43.222, O43.223, O43.229, O43.231, O43.233, O43.239, O43.819, O43.892, O43.893, O43.899, O43.90, O44.00, O44.01, O44.02, O44.03, O44.11, O44.20, O44.21, O44.23, O44.32, O44.40, O44.41, O44.52, O45.003, O45.011, O45.012, O45.021, O45.029, O45.093, O45.8X1, O45.8X2, O45.8X3, O45.90, O45.91, O45.93, O46.001, O46.011, O46.021, O46.029, O46.091, O46.093, O46.8X1, O46.8X3, O46.8X9, O47.00, O47.03, O47.1, O47.9, O48.0, O48.1, O60.00, O60.02, O60.10X0, O60.10X2, O60.10X5, O60.12X1, O60.12X4, O60.12X5, O60.12X9, O60.13X0, O60.13X1, O60.14X0, O60.14X2, O60.20X1, O60.20X2, O60.20X3, O60.20X5, O60.20X9, O60.22X1, O60.22X4, O60.22X9, O60.23X4, O60.23X5, O61.0, O61.9, O62.0, O62.1, O62.2, O62.3, O62.4, O62.9, O63.0, O64.0XX1, O64.0XX2, O64.0XX3, O64.0XX4, O64.0XX5,

	O64.0XX9, O64.1XX0, O64.1XX1, O64.1XX2, O64.1XX4, O64.1XX9, O64.2XX0, O64.2XX1, O64.2XX2, O64.2XX3, O64.2XX4, O64.3XX0, O64.3XX1, O64.3XX2, O64.3XX3, O64.3XX5, O64.3XX9, O64.4XX0, O64.4XX1, O64.4XX3, O64.4XX5, O64.5XX3, O64.5XX4, O64.5XX9, O64.8XX2, O64.8XX3, O64.8XX4, O64.8XX5, O64.8XX9, O64.9XX0, O64.9XX1, O64.9XX4, O65.2, O65.8, O65.9, O66.0, O66.3, O66.40, O66.5, O66.6, O66.8, O66.9, O67.9, O69.0XX2, O69.0XX3, O69.0XX5, O69.1XX2, O69.1XX3, O69.1XX4, O69.1XX5, O69.1XX9, O69.2XX0, O69.2XX2, O69.2XX3, O69.2XX5, O69.2XX9, O69.3XX1, O69.3XX3, O69.3XX5, O69.3XX9, O69.4XX0, O69.4XX1, O69.4XX3, O69.5XX1, O69.5XX2, O69.5XX9, O69.81X0, O69.81X1, O69.81X5, O69.81X9, O69.82X2, O69.82X3, O69.82X5, O69.89X2, O69.89X3, O69.89X4, O69.89X5, O69.89X9, O69.9XX0, O69.9XX2, O69.9XX4, O70.0, O70.1, O70.2, O70.23, O70.9, O71.00, O71.03, O71.1, O71.2, O71.4, O71.5, O71.9, O72.1, O72.2, O72.3, O74.1, O74.2, O74.3, O74.6, O74.7, O74.8, O74.9, O75.2, O75.4, O75.81, O75.82, O77.0, O77.9, O80, O82, O85, O86.0, O86.01, O86.03, O86.04, O86.09, O86.12, O86.29, O86.89, O87.2, O87.3, O87.4, O88.019, O88.111, O88.113, O88.13, O88.212, O88.219, O88.22, O88.23, O88.311, O88.319, O88.32, O88.33, O88.811, O88.812, O88.819, O88.82, O88.83, O89.01, O89.1, O89.2, O89.5, O90.0, O90.2, O90.4, O90.89, O90.9, O91.011, O91.012, O91.019, O91.02, O91.03, O91.112, O91.119, O91.13, O91.211, O91.212, O91.22, O91.23, O92.019, O92.03, O92.111, O92.112, O92.20, O92.29, O92.3, O92.4, O92.5, O92.70, O94, O98.019, O98.111, O98.112, O98.12, O98.211, O98.212, O98.219, O98.311, O98.312, O98.313, O98.32, O98.411, O98.412, O98.419, O98.511, O98.513, O98.53, O98.612, O98.73, O98.811, O98.812, O98.813, O98.819, O98.82, O98.83, O99.019, O99.02, O99.03, O99.119, O99.12, O99.13, O99.210, O99.213, O99.214, O99.280, O99.282, O99.283, O99.310, O99.311, O99.312, O99.314, O99.315, O99.321, O99.322, O99.324, O99.331, O99.332, O99.334, O99.335, O99.343, O99.345, O99.350, O99.351, O99.352, O99.412, O99.419, O99.42, O99.43, O99.512, O99.53, O99.613, O99.62, O99.63, O99.711, O99.719, O99.72, O99.73, O99.820, O99.825, O99.840, O99.841, O99.843, O99.845, O99.891, O99.892, O9A.119, O9A.212, O9A.219, O9A.22, O9A.312, O9A.32, O9A.33, O9A.412, O9A.511, O9A.512, O9A.52, O9A.53, Z33.2, Z33.3, Z34.00, Z34.01, Z34.02, Z34.80, Z34.83, Z36.0, Z36.2, Z36.81, Z36.82, Z36.83, Z36.85, Z36.86, Z36.9, Z37.0, Z37.3, Z37.4, Z37.50, Z37.51, Z37.52, Z37.53, Z37.59, Z37.60, Z37.64, Z37.9, Z39.0, Z39.2, Z3A.00, Z3A.01, Z3A.10, Z3A.11, Z3A.12, Z3A.13, Z3A.14, Z3A.15, Z3A.16, Z3A.17, Z3A.19, Z3A.21, Z3A.22, Z3A.24, Z3A.30, Z3A.35, Z3A.36, Z3A.38, Z3A.39, Z3A.42
History of smoking/tobacco use	The occurrence of a medical claim with the following attributes during the baseline period:<br>Diagnosis Code, ICD-10 is any of: Z71.6, Z72.0, Z87.891, F17.21, F17.210, F17.211, F17.213, F17.218, F17.219, F17.22, F17.220, F17.221, F17.223, F17.228, F17.229, F17.29, F17.290, F17.291, F17.293, F17.298, F17.299, T65.21, T65.211, T65.211A, T65.211D, T65.211S, T65.212, T65.212A, T65.212D, T65.212S, T65.213, T65.213A, T65.213D, T65.213S, T65.214, T65.214A, T65.214D, T65.214S, O99.33, O99.330, O99.331, O99.332, O99.333, O99.334, O99.335<br>OR<br>Procedure Code, HCPCS and CPT is any of: 1034F, 99406, 99407, 4001F, 4004F, G0436, G0437, S4995, G9016, G9458, S9453, G9276<br>OR<br>The occurrence of Pharmacy Claims with the following attributes during the baseline period:<br>Generic Name is any of: NICOTINE, NICOTINE BITARTRATE, NICOTINE POLACRILEX, VARENICLINE TARTRATE
Stroke or cerebrovascular disease	The occurrence of a medical claim with the following attributes during the baseline period:<br>Diagnosis Code, ICD-10 is any of: G46.x, I63.x
Tuberculosis	The occurrence of medical claim with the following attributes during the baseline period:<br>Diagnosis Code, ICD-10 is any of: A15.x-A19.x

Abbreviations: COPD, chronic obstructive pulmonary disease; COVID-19, coronavirus disease 2019; CPT, Current Procedural Terminology; HCPCS, Healthcare Common Procedure Coding System; HIV, human immunodeficiency virus; ICD-10, International Classification of Diseases, Tenth Revision.

eTable 5. Subgroup definitions

Health status subgroups[a]	
**Subgroup**	**Definition**
Immunocompromised condition subgroup	Individuals were included in this subgroup if they had ≥1 immunocompromised condition. Immunocompromised conditions of interest are listed in eTable 4.
Vulnerable condition subgroup	Individuals were included in this subgroup if they had ≥1 vulnerable condition and did not have an immunocompromised condition. Vulnerable conditions of interest are listed in eTable 4.
Other healthy population subgroup	Individuals were included in this subgroup if they did not have an immunocompromised or vulnerable condition.
**Vaccination status subgroups**	
**Subgroup**	**Definition**
Vaccinated	Evidence of receiving at least one dose of the Pfizer-BioNTech COVID-19 vaccine, Moderna COVID-19 vaccine, or Johnson & Johnson/Janssen COVID-19 vaccine.
Unvaccinated (ie, no evidence of vaccination)	No evidence of receiving the Pfizer-BioNTech COVID-19 vaccine, Moderna COVID-19 vaccine, or Johnson & Johnson/Janssen COVID-19 vaccine.

[a] The immunocompromised condition, vulnerable condition, and other healthy population subgroups are mutually exclusive.

Abbreviations: COVID-19, coronavirus disease 2019.

eTable 6. Definitions of higher and lower SARS-CoV-2 spike-protein targeted antibody levels

**Assay**	**Equation**	**Antibody test result value equal to 250 BAU/ml**
DiaSorin (Trimeric S) IgG	BAU = 2.6 * (TrimericS assay value)	96.15 AU/mL
Roche Diagnostics (Cov-2 spike)	BAU = (Cov-2 spike assay value) / 0.972	243 units/mL
Siemens Healthineers Diagnostics (COV2G) IgG	BAU = 7.5145(COV2G assay value)^2 + 35.379(COV2G value) + 18.337	3.68 index units
Siemens Healthineers Diagnostics (sCOVG) IgG	BAU = 45.078 * [(sCOVG assay value)$^{0.7984}$]	8.55 index units

Because each semi-quantitative assays has a different measurement scale, all antibody test result values were converted to WHO BAU/mL. Lower antibody levels were defined as a value of <250 BAU/mL and higher antibody levels were defined as values ≥250 BAU/mL. Conversion to BAU/mL based on: 1) Freeman J, Conklin J. Standardization of two SARS-CoV-2 serology assays to the WHO 20/136 human standard reference material. J Virol Methods. 2022 Feb;300:114430. doi: 10.1016/j.jviromet.2021.114430. Epub 2021 Dec 13. PMID: 34915088; PMCID: PMC8667347, 2) Internal communications from DiaSorin (DiaSorin customer information letter, February 2, 2021), and 3) Internal communications from Roche Diagnostics (Department of Research & Development, for centralized and point of care solutions, January 12, 2021).

Abbreviations: AU, Arbitrary units; BAU, binding antibody units; SARS-CoV-2, severe acute respiratory syndrome coronavirus 2.

eTable 7. Characteristics of individuals with higher and lower SARS-CoV-2 spike-protein targeted antibody levels

Characteristic	Unmatched			Matched		
	Higher antibody level n = 102,866	Lower antibody level n = 39,837	Std diff[a]	Higher antibody level n = 39,740	Lower antibody level n = 39,740	Std diff[a]
**Year/season of index**			0.09			0.02
Winter 2020-2021 [b]	674 (0.7%)	550 (1.4%)		494 (1.2%)	524 (1.3%)	
Spring 2021 [b]	16,223 (15.8%)	6,447 (16.2%)		6,310 (15.9%)	6,439 (16.2%)	
Summer 2021 [b]	31,534 (30.7%)	11,179 (28.1%)		10,964 (27.6%)	11,169 (28.1%)	
Fall 2021 [b]	40,723 (39.6%)	16,483 (41.4%)		16,644 (41.9%)	16,436 (41.4%)	
Winter 2021[b]	13,712 (13.3%)	5,178 (13.0%)		5,328 (13.4%)	5,172 (13.0%)	
**Age (years)**	52.4 ± 15.5	49.6 ± 15.7	0.18	49.7 ± 15.7	49.6 ± 15.7	0.01
**Female**	62,536 (60.8%)	23,982 (60.2%)	0.01	23,926 (60.2%)	23,930 (60.2%)	0.00
**Region**			0.17			0.02
Midwest	7,413 (7.2%)	4,317 (10.8%)		4,062 (10.2%)	4,236 (10.7%)	
South	37,201 (36.2%)	14,376 (36.1%)		14,572 (36.7%)	14,372 (36.2%)	
West	13,191 (12.8%)	6,183 (15.5%)		6,326 (15.9%)	6,173 (15.5%)	
Northeast	45,041 (43.8%)	14,960 (37.6%)		14,779 (37.2%)	14,958 (37.6%)	
Other or unknown	20 (0.0%)	1 (0.0%)		1 (0.0%)	1 (0.0%)	
**SNF or nursing home utilization**	846 (0.8%)	268 (0.7%)	0.02	168 (0.4%)	268 (0.7%)	0.03
**Had ≥ 1 immunocompromising condition**	7,571 (7.4%)	2,866 (7.2%)	0.01	2,662 (6.7%)	2,846 (7.2%)	0.02
**Had ≥ 1 vulnerable condition**	51,564 (50.1%)	17,268 (43.3%)	0.14	17,257 (43.4%)	17,256 (43.4%)	0.00
**COVID-19 vaccination status**			0.46			0.01
Fully vaccinated plus a booster	1,031 (1.0%)	35 (0.1%)		35 (0.1%)	35 (0.1%)	
Fully vaccinated	29,925 (29.1%)	5,411 (13.6%)		5,427 (13.7%)	5,411 (13.6%)	
Partially vaccinated	8,092 (7.9%)	1,771 (4.4%)		1,693 (4.3%)	1,771 (4.5%)	
Unvaccinated	63,818 (62.0%)	32,620 (81.9%)		32,585 (82.0%)	32,523 (81.8%)	

Values presented as number (%) for categorical variables and as mean ± standard deviation for continuous variables.

Higher and lower defined as ≥250 BAU/mL and <250 BAU/mL, respectively, based on studies supporting this general threshold: 1) Conseil d'Orientation de la Stratégie Vaccinale Recommandations pour la protection des personnes sévèrement immunodéprimées contre le Covid-19 (Vaccination et prophylaxie primaire) – 19 Novembre 2021. https://solidarites-sante.gouv.fr/IMG/pdf/cosv_-_recommandations_pour_la_protection_des_personnes_severement_immunodeprimees_-_19_novembre_2021.pdf. Accessed December 12, 2022. 2) Piñana JL, López-Corral L, Martino R, et al. SARS-CoV-2 vaccine response and rate of breakthrough infection in patients with hematological disorders. *J Hematol Oncol*. 2022;15(1):54. and 3) Feng S, Phillips DJ, White T, et al. Correlates of protection against symptomatic and asymptomatic SARS-CoV-2 infection. *Nat Med*. 2021;27(11):2032-2040. Conversion to BAU/mL based on : 1) Freeman J, Conklin J. Standardization of two SARS-CoV-2 serology assays to the WHO 20/136 human standard reference material. J Virol Methods. 2022 Feb;300:114430. doi: 10.1016/j.jviromet.2021.114430.

Epub 2021 Dec 13. PMID: 34915088; PMCID: PMC8667347, 2) Internal communications from DiaSorin (DiaSorin customer information letter, 2 February 2021), and 3) Internal communications from Roche Diagnostics (Department of Research & Development, for centralized and point of care solutions, January 12, 2021).

[a] Standardized difference > 0.10 represents a meaningful imbalance between exposure groups.
[b] Winter 2020-2021 was defined as 1 December 2022 to 28 February 2021. Spring 2021 was defined as 1 March 2021 to 31 May 2021. Summer 2021 was defined as 1 June 2021 to 31 August 2021. Fall 2021 was defined as 1 September 2021 to 31 November 2021. Winter 2021 was defined as 1 December 2021 to 31 December 2021.

Abbreviations: COVID-19, coronavirus disease 2019; SARS-CoV-2, severe acute respiratory syndrome coronavirus 2; SNF, skilled nursing facility; std diff, standardized difference.
